# Cell Death and Senescence‐Based Molecular Classification and an Individualized Prediction Model for Lung Adenocarcinoma

**DOI:** 10.1002/mco2.70237

**Published:** 2025-05-29

**Authors:** Pan Wang, Chaoqi Zhang, Peng Wu, Zhihong Zhao, Nan Sun, Qi Xue, Shugeng Gao, Jie He

**Affiliations:** ^1^ Department of Thoracic Surgery National Cancer Center/National Clinical Research Center for Cancer/Cancer Hospital, Chinese Academy of Medical Sciences and Peking Union Medical College Beijing China

**Keywords:** cancer subtype, cellular senescence, cell death, lung adenocarcinoma, tumor microenvironment

## Abstract

The exploration of cell death and cellular senescence (CDS) in cancer has been an area of interest, yet a systematic evaluation of CDS features and their interactions in lung adenocarcinoma (LUAD) to understand tumor heterogeneity, tumor microenvironment (TME) characteristics, and patient clinical outcomes is previously uncharted. Our study characterized the activities and interconnections of 21 CDS features in 1788 LUAD cases across 15 cohorts, employing unsupervised clustering to categorize patients into three CDS subtypes with distinct TME profiles. The CDS index (CDSI), derived from principal component analysis, was developed to assess individual tumor CDS regulation patterns. Twelve CDSI core genes, enriched in proliferating T cells within the TME as per single‐cell analysis, were identified and their functional roles and prognostic significance were validated. High CDSI correlated with improved overall survival in discovery cohort, four independent validation cohorts, and subgroup analysis. CDSI‐low patients exhibited a favorable clinical response to immunotherapy and potential sensitivity to mitosis pathway drugs, while CDSI‐high patients might benefit from drugs targeting ERK/MAPK and MDM2–p53 pathways. The clinical utility of CDSI was further validated using 9185 pan‐cancer samples, demonstrating the broad relevance of our prediction model across various cancer types and its potential clinical implications for cancer management.

## Graphical abstract

The study systematically investigated 21 cell death and cellular senescence (CDS) features in lung adenocarcinoma, including their expressions, interactions, and immuno‐oncology profiles. Three CDS subtypes were identified to generate a CDS index (CDSI) for personalized regulation patterns. The CDSI's predictive power for patient prognosis and drug response was validated across multiple cohorts and further assessed in pan‐cancers.

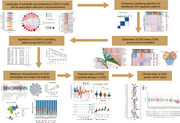

## 1 Introduction

Lung cancer remains a leading cause of cancer‐related deaths worldwide [1], with lung adenocarcinoma (LUAD) accounting for over 40% of cases [2]. Despite steady progress in therapeutic strategies, the 5‐year average survival rate of patients with lung cancer remains suboptimal [3]. Treatment failure can be largely attributed to substantial heterogeneity within the tumor [4].

Cell death is essential for homeostasis. Historically, there are two primary forms of cell death: “programmed” apoptosis and “accidental” necrosis [5, 6]. However, studies have revealed contextually distinct cell death modes, most of which are governed by genetic molecular mechanisms, implying that they are types of regulated cell death (RCD) [7]. To date, over 20 RCD types have been described [7]. Although the cell morphology and functions of these RCD modes differ, considerable crosstalk exists among RCD modes during the initiation, signaling, and execution of cell death [8]. Cells can “program” their death to tailor immune responses (immunogenic cell death, ICD). RCD can be a consequence of immune responses [9]. Although RCD dysregulation is associated with various cancers, how these RCD modes are selectively activated in cancer cells and how the effector response to RCD is defined by signals within both the dying cell and the environment remain unexplored [10].

Cellular senescence (CS) is characterized by specific morphological traits, deregulated metabolism, and activation of a senescence‐associated secretory phenotype [11]. Senescence‐inducing signals can identify different types of CS [12]. CS protects against neoplasia; however, senescent cells may promote tumor development and progression [13]. Moreover, CS, together with other aging hallmarks, defines an aging microenvironment [14], which promotes highly aggressive and metastatic diseases.

RCD and CS are stress responses. Depending on the type, magnitude, and duration of stress, cells determine their fate by inducing repair, cell death, or senescence, implying a functional link between RCD and CS [9, 13]. Apoptosis, autophagy, and necroptosis are associated with senescence [15, 16, 17]. Previous studies on relationships between cell death and cellular senescence (CDS) and cancer have been confined to one or a few widely studied RCD modes or to CS as a whole [18, 19, 20, 21, 22]. However, to the best of our knowledge, no study has systematically documented the interactions between RCD and CS, their collective impact on defining cancer cell characteristics, and the heterogeneity of the tumor microenvironment (TME).

In this study, we analyzed the activities of 21 CDS features (Table S1), including 13 RCD modes, four cell death‐related signaling pathways, and four CS types, across 1788 LUAD cases from 15 different cohorts (Table s2). The entire workflow is outlined in Figure S1. Our findings offer insights into the generation of tumor heterogeneity in LUAD through CDS features and their crosstalk, the influence of CDS regulation patterns on the TME, and the impact of CDS features on patient prognosis.

## 2 Results

### 2.1 Modular Landscape of CDS Activities in LUAD

**FIGURE 1:**
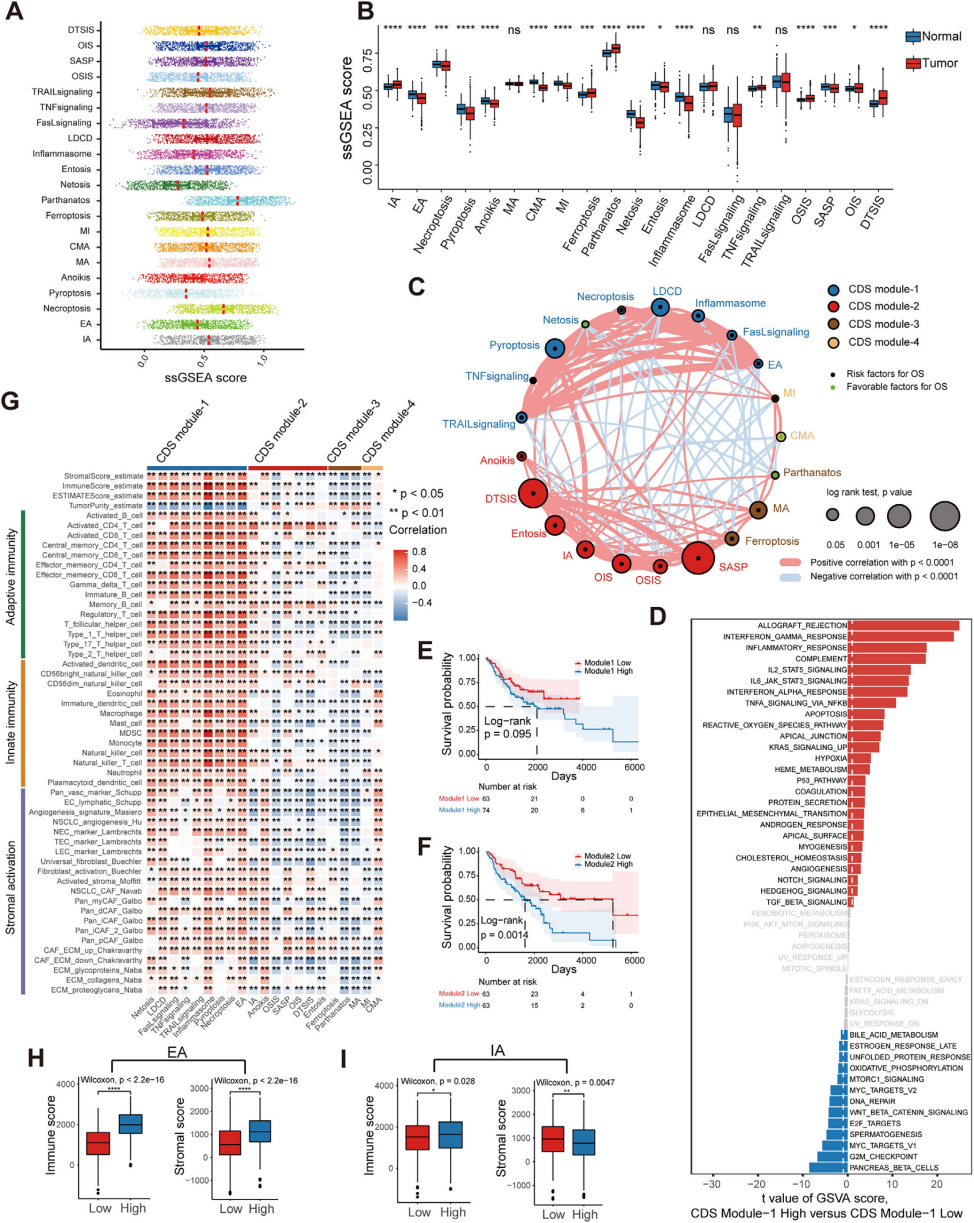
Landscape of CDS activities and related biological processes based on the LuMMD. (A) Distribution of the CDS activities in the LUAD samples. (B) Differences in the CDS activities between LUAD and normal lung samples. (C) Interactions between CDS features in LUAD. The circle size corresponds to the survival impact of each CDS; calculation used the formula log_10_ (log‐rank test *p* values indicated). Green dots in the circle indicate risk factors; black dots in the circle indicate protective factors. The lines connecting CDS features represent their interactions. The thickness of the line represents the strength of correlation estimated using Spearman correlation analysis. Positive correlation is indicated in red and negative correlation in blue. (D) Differences in pathway activities scored per patient using GSVA between CDS module‐1 high and low LUADs. Shown are *t*‐values from a linear model. (E and F) Kaplan–Meier curves of OS for patients with LUAD stratified by activities of module‐1 (E) and ‐2 (F). *p* Value was calculated using the log‐rank test. (G) Correlation between each CDS feature and each TME‐infiltrating cell type using Spearman analyses. Positive and negative correlations are indicated in red and blue, respectively. (H and I) Differences in immune and stromal scores between LUAD samples with different activities of (H) EA and (I) IA. In (B) and (H and I), the upper and lower ends of the boxes represent interquartile range of values. The lines in the boxes represent median values, and black dots show outliers. *p* Values were determined using Wilcoxon rank‐sum and Kruskal–Wallis tests. ns, not significant; *p < 0.05; **p ≤ 0.01; ***p ≤ 0.001; ****p ≤ 0.0001.

All CDS features of LUAD demonstrated a wide range of values, and the majority showed significant differences in expression between tumor and normal samples (Figures 1A,B and S2A,B). No CDS activity differences were observed between LUAD subgroups based on sex, age, smoking status, or stage (Figure S2C–J). Univariate Cox regression analysis of the LUAD merged microarray‐acquired dataset (LuMMD) revealed that most CDS features predicted unfavorable prognoses (Table S3). We constructed a network depicting the landscape of CDS interactions and their prognostic significance in LUAD, identifying four distinct CDS modules (Figures 1C and S3A and Table S4). Three‐quarters of the CDS features had over 10 significant associations (Spearman's *r* < 0.0001), with intrinsic apoptosis (IA) being the most connected. Positive correlations (72%) outnumbered negative correlations (28%). The interactions between four CS and 17 cell death‐related features accounted for 31.3% of all interactions. Highly correlated CDS pairs (Spearman's *r* > 0.4) were predominantly in module‐1 and module‐2. Module‐1 contained typical ICD modes, such as extrinsic apoptosis (EA), pyroptosis, and necroptosis, and was associated with immune response according to gene set variation analysis (GSVA) enrichment analysis (Figure 1D). Module‐2, based on the CS features, was enriched in cell cycle pathways like E2F_targets and G2M_checkpoints (Table S5). Kaplan–Meier (KM) analysis revealed that module‐1, ‐2, and ‐3 negatively impacted prognosis, whereas module‐4 had no significant effect on survival (Figures 1E,F and S3B,C).

### 2.2 Heterogeneity of CDS Shapes the TME in LUAD

Correlation analysis of the LuMMD and The Cancer Genome Atlas (TCGA) datasets revealed a strong association between CDS features and tumor‐infiltrating immune and stromal cells (Figures 1G and S3D). Module‐1 showed a positive correlation with most TME‐infiltrating cells, while other modules displayed greater heterogeneity in TME regulation. For example, in module‐2, anoikis was positively correlated with angiogenesis, whereas the other features exhibited an inhibitory effect on angiogenesis (Figure 1G). Conceptually related CDS features (e.g., EA and IA) had distinct effects on the TME. The EA‐high group had substantially higher immune and stromal scores than the EA‐low group (Figure 1H). The IA‐high group had a higher immune score but lower stromal score than the IA‐low group (Figure 1I). Similar findings were obtained by comparing the regulatory effects of different autophagy modes on the TME (Figure S3E–G).

### 2.3 CDS Regulation Patterns in LUAD

**FIGURE 2:**
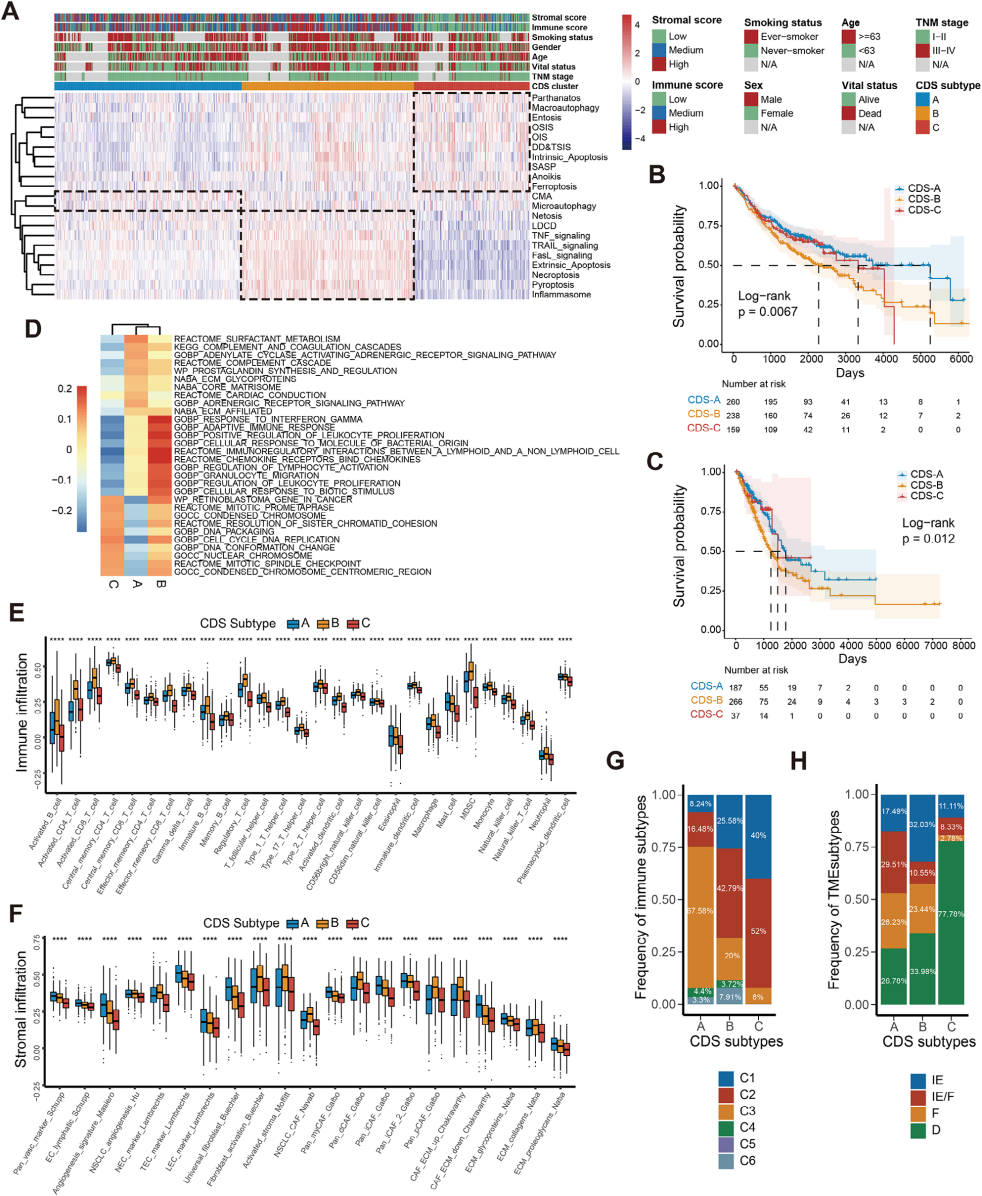
Clustering for CDS regulation patterns, clinicopathological features, biological functions, and TME characteristics of each pattern. (A) Unsupervised clustering of CDS features for LUAD in the LuMMD cohort. Immune and stromal scores, smoking status, sex, age, vital status, TNM stage, and CDS cluster group are shown as patient annotations. (B and C) Kaplan–Meier curves of OS for three CDS subtypes of patients in the (B) LuMMD and (C) TCGA cohorts. *p* Values were calculated using the log‐rank test. (D) GSEA reveals distinct enriched gene sets between CDS subtypes. In the heatmap, rows are defined by the selected 30 gene sets, and columns by GSVA scores for each subtype. (E and F) TME‐infiltrating (E) immune, and (F) stromal cells in three CDS subtypes based on the LuMMD cohort. The upper and lower ends of the boxes represent interquartile range of values. The lines in the boxes represent median values, and black dots show outliers. *p* Values were determined using the Kruskal–Wallis test; ****p ≤ 0.0001. (G and H) Proportion of pan‐cancer (G) immune subtypes and (H) conserved microenvironment subtypes in three CDS subtypes. IE, immune‐enriched; IE/F, immune‐enriched, fibrotic; F, fibrotic; D, deserted.

Unsupervised clustering of CDS profiles from 817 LuMMD tumor samples stratified patients into three CDS subtypes (CDS‐A, ‐B, ‐C) (Figure S4A and Table S6). Figure 2A shows the relationships between CDS subtypes and modules. CDS‐A was marked by high module‐4 activity and low module‐2 activity, with intermediate activities in module‐1 and ‐3. CDS‐B featured high module‐1 and ‐2 activation, and moderate activities in module‐3 and ‐4. CDS‐C was characterized by high module‐2 and ‐3 activities and low module‐1 and ‐4 activities. The distributions of CDS features among CDS subtypes are illustrated in Figure S4B. CDS‐C had the highest levels of CS features, whereas CDS‐B was enriched in ICD modes. Ferroptosis was activated in CDS‐C but repressed in CDS‐A. CDS‐A had the best survival prognosis, whereas CDS‐B had the worst (log‐rank test, *p* = 0.0067; Figure 2B). These results were validated in TCGA cohort (Figures 2C and S4C and Table S7). A pathway heatmap based on gene set enrichment analysis (GSEA) highlighted that CDS‐A was enriched for complement activation and extracellular matrix (ECM) modulation pathways, CDS‐B for immune response pathways, and CDS‐C for cell cycle pathways (Figure 2D and Table S8). Subsequent TME characterization in both datasets revealed that CDS‐B had the highest infiltration for almost all TME cell types, except for eosinophils, mast cells, endothelial cells, and universal fibroblasts, which were more abundant in CDS‐A. CDS‐C exhibited a desert‐like TME with the lowest immune and stromal infiltration (Figures 2E,F and S4D,E).

We further assessed the previously published TME‐based subtypes in relation with our subtyping methodology [23, 24]. Concerning the pan‐cancer immune subtypes [23], CDS‐A exhibited a predominance of the “inflammatory” subtype associated with the most favorable prognosis, whereas CDS‐C was primarily composed of the “wound healing” and “IFN‐γ” subtypes, both characterized by high proliferation rates (Figure 2G). Regarding the conserved pan‐cancer microenvironment subtypes [24], CDS‐A encompassed a higher proportion of patients displaying the “immune‐enriched, fibrotic” and “fibrotic” phenotypes, characterized by angiogenesis and fibroblast enrichment. CDS‐C was dominated by the “depleted” subtype, marked by a high proliferation rate and low immune and stromal cell infiltration (Figure 2H).

### 2.4 Generation of the CDS Index and Functional Annotation

**FIGURE 3:**
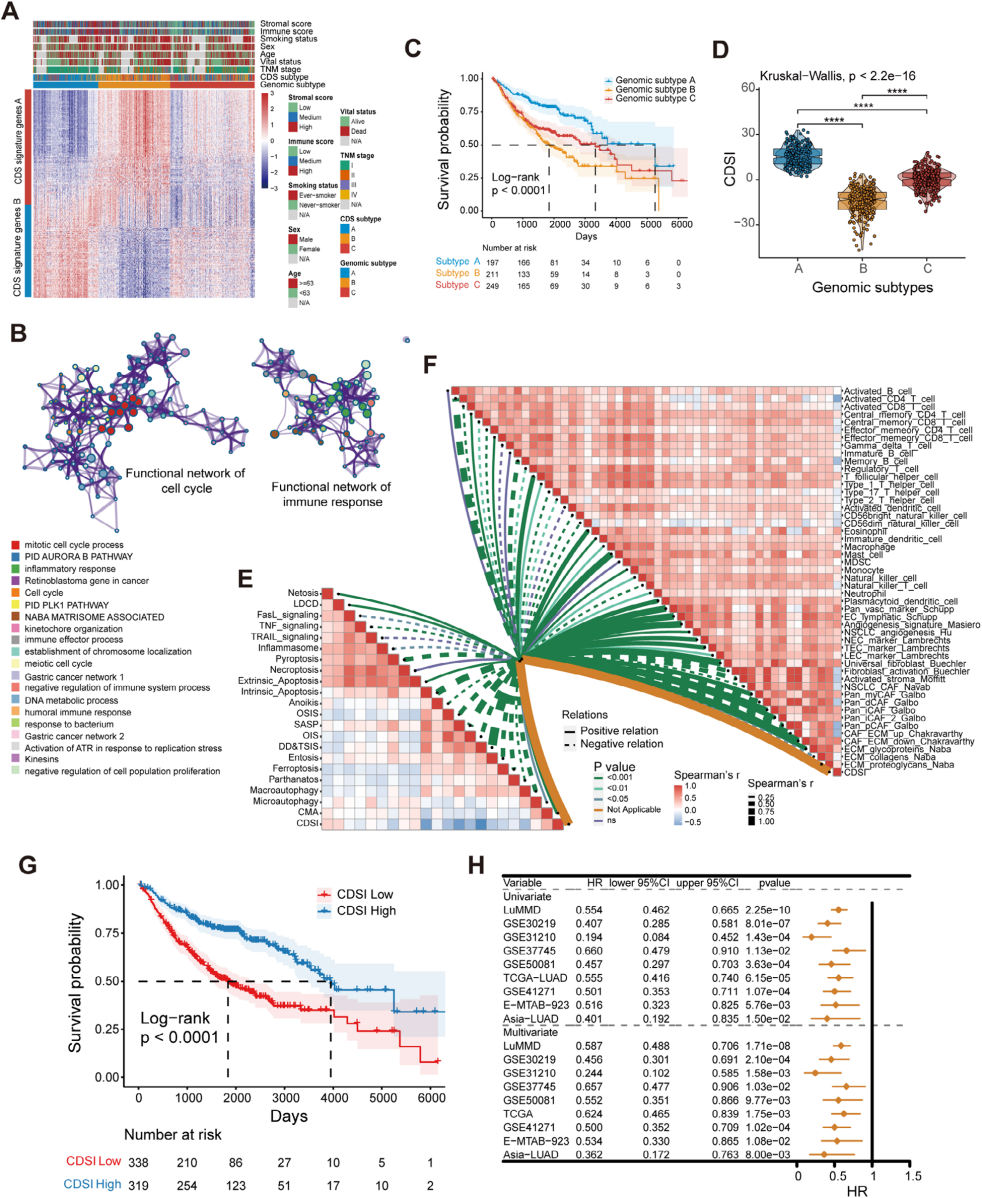
Generation of the CDSI and its prognostic potential. (A) Unsupervised clustering of CDS‐associated DEGs to classify patients into three genomic subtypes: gene clusters (A–C). Immune and stromal scores, smoking status, sex, age, vital status, TNM stage, CDS cluster, and gene cluster group are shown as patient annotations. (B) Visualization of the functional enrichment network of the CDS signature genes, in which pathways with similar functions are clustered and labeled with the same color. (C) Kaplan–Meier curves of OS for three genomic subtypes of patients in the LuMMD cohort. The *p* value was calculated using the log‐rank test. (D) Distribution of CDSI in CDS genomic subtypes visualized using box‐violin plots. The upper and lower ends of the boxes represent the interquartile range of values, the lines in the boxes represent median values, and black dots show outliers. *p* Values were tested using the Kruskal–Wallis test. ****p ≤ 0.0001. (E) Correlations between CDSI and CDS activities. (F) Correlations between CDSI and TME‐infiltrating immune and stromal cell types. (G) Kaplan–Meier curve of OS for the CDSI‐high and ‐low patients based on the LuMMD cohort. The *p* value was calculated using the log‐rank test. (H) Forest plot showing univariate and multivariate Cox regression analyses of CDSI associated with major clinicopathological features of different cohorts.

Unsupervised clustering of 559 differentially expressed genes (DEGs) across CDS subtypes in LuMMD patients identified three genomic subtypes (Figure S5A,B), which aligned with our previous CDS feature clustering (Figure 3A). Dimension reduction yielded 245 signature genes, comprising 136 and 109 genes from CDS gene signatures A and B, respectively (Table S9). Functional annotation of these signature genes using Metascape highlighted enrichment in pathways related to mitotic cell cycle and inflammatory response (Figure 3B and Table S10) [25]. The genomic subtypes mirrored the CDS subtypes in terms of CDS features activities, TME cell infiltration patterns, and prognostic outcomes (Figures 3C and S5C–E).

We developed a CDS Index (CDSI) from the CDS gene signatures to individually quantify CDS regulation in each patient. The Kruskal–Wallis test revealed a significant difference in CDSI across CDS genomic subtypes (*p* < 2.2e−16; Figure 3D). The CDSI inversely correlated with most CDS features, particularly DNA damage/telomere stress‐induced senescence and IA (Figure 3E). It positively correlated with the infiltration of endothelial cells, universal fibroblasts, and innate immune cells, such as mast cells and eosinophils, but negatively with effector cells, cancer‐associated fibroblasts (CAFs), natural killer cells, and M1 macrophages (Figure 3F). Validation using nine independent algorithms confirmed these findings (Figures S6–S13). Cancer hallmarks profiling indicated that CDSI was negatively correlated with E2F_targets, G2M_checkpoint, and MYC_targets, and positively with bile_acid_metabolism, heme_metabolism, and UV_response_DN (Figure S14).

### 2.5 CDSI is an Independent Prognostic Feature in LUAD

The observed associations between CDSI and immuno‐oncological features motivated us to investigate its clinical significance in LUAD. Patients were stratified into CDSI‐high and ‐low groups based on the optimal cutoff point to assess the prognostic value of CDSI (Table S11). Figure S15A illustrates the correlations among CDS‐defined clusters, CDSI‐defined subgroups, and survival status in the LuMMD cohort. The CDSI‐high group had significantly better overall survival (OS) than the CDSI‐low group (median survival time: 3949 vs. 1839 days, respectively; log‐rank test, *p* < 0.0001; Figure 3G), with a similar trend in disease‐free survival (Figure S15B). Stratified analysis of major clinicopathological parameters including sex, age, smoking status, *EGFR* status, and tumor‐node‐metastasis (TNM) stage confirmed a survival benefit for the CDSI‐high group (log‐rank test, *p* < 0.01; Figure S15C–G). To validate the reproducibility of our model, we conducted independent experiments in three international cohorts (TCGA–LUAD, GSE41271, and E‐MTAB‐923) and one Chinese cohort (LUAD‐Singapore). KM analyses revealed a significantly higher mortality risk for the CDSI‐low group (log‐rank test, *p* < 0.01; Figure S15H–K). Univariate analysis indicated that a higher CDSI was associated with a better OS (hazard ratio [HR] 0.194–0.660); after adjusting for major clinicopathologic factors, CDSI emerged as an independent indicator of better OS in LUAD across all datasets (HR 0.244–0.657; Figure 3H and Table S12). To assess the predictive ability of the CDSI with that of the gold‐standard TNM stage, we employed a Cox regression model that integrated clinicopathological parameters with either TNM stage or CDSI to forecast postoperative survival in LUAD patients over 2000 days. The CDSI model outperformed the TNM model, as evidenced by an NRI score of 0.131 for the LuMMD cohort and 0.137 for TCGA cohort (Figure S15L,M).

### 2.6 Molecular Characterization of the CDSI Phenotypes

**FIGURE 4:**
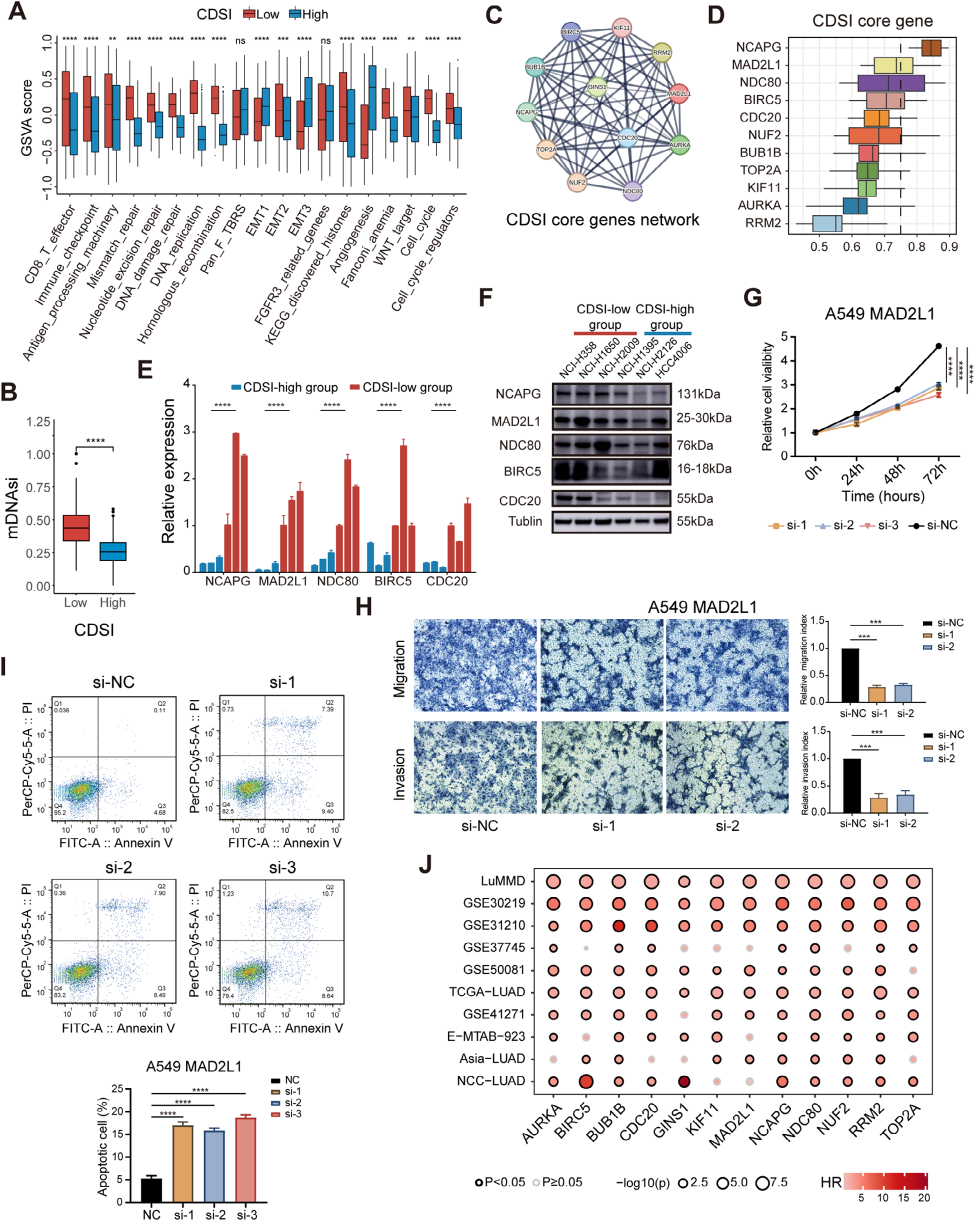
Molecular characterization of CDSI phenotypes. (A–C) Differences in (A) CDS activities, and (B) mRNAsi score between the CDSI‐high and ‐low groups. The upper and lower ends of the boxes represent interquartile range of values. The lines in the boxes represent median values, and black dots show outliers. *p* Values were calculated using the Wilcoxon rank‐sum test. ns, not significant; **p ≤ 0.01; ***p ≤ 0.001; ****p ≤ 0.0001. (C) Protein–protein interaction network of the CDSI core genes. (D) Boxplots showing the distribution of functional similarities among the CDSI core genes. The right and left ends of the boxes represent interquartile range of values, the lines in the boxes represent mean values, and the dashed line represents the cutoff value of 0.75. (E) Comparison of relative level of selected CDSI core genes in different CDSI cell subsets by qRT‐PCR. (F) Western blotting of the selected CDSI core genes in LUAD cell lines of different CDSI subgroups. (G–I) CCK‐8 assay (G), Transwell assay (H), and cell apoptosis analysis (I) performed in A549 cell line transfected with control siRNA or *MAD2L1* siRNA. (J) Summary of Kaplan–Meier analysis results across datasets. The size of the circle represents significance; calculation used the formula ‐log_10_ (log‐rank test *p* values indicated).

The CDSI‐high group displayed reduced RCD and CS features but showed higher levels of NETosis, lysosome‐dependent cell death, and EA than the CDSI‐low group (Figure S16A). CDS feature interactions remained largely stable across the CDSI phenotypes, with minor localized alterations (Figure S16B and Table S13). CDSI‐high was linked to increased angiogenesis, whereas CDSI‐low was associated with enhanced activation of cell cycle, DNA damage response, and CD8 T effector pathways (Figure 4A), as well as higher cancer stemness levels, as measured by the mRNAsi score (Figure 4B) [26]. To characterize the molecular features of CDSI, we analyzed the differential expression of 704 CRISPR‐screened essential genes from the DepMap portal [27], and identified 12 CDSI core genes (Figure S16C). Independent genome‐scale CRISPR‐Cas9 fitness screens confirmed the essentiality of these genes, with 10 identified as pan‐cancer core fitness genes and 11 as lung cancer core fitness genes (Table S14) [28]. STRING analysis revealed a cohesive protein–protein interaction network among these genes (enrichment *p* < 1.0e−16; Figure 4C) [29], with *NCAPG* emerging as the hub gene by ranking the average functional similarity among its members (Figure 4D) [30]. We verified the association between CDSI and its core genes by analyzing gene expressions of the top genes in the network in cell lines with varying CDSI levels (Table S15). Six ATCC‐sourced LUAD cell lines from the Cancer Cell Line Encyclopedia (CCLE) (CDSI‐high: NCI‐H2009, NCI‐H1650, NCI‐H358; CDSI‐low: NCI‐H1395, NCI‐H2126, HCC4006) were collected. Quantitative reverse‐transcription PCR (qRT‐PCR) and western blotting revealed a significant upregulation of these genes in the CDSI‐low cell lines compared with the CDSI‐high cell lines (Figures 4E,F and S17A). Importantly, 10 of the 12 CDSI core genes have been experimentally validated for their tumor‐promoting effects in LUAD (Table S16). We further explored the functions of *MAD2L1* and *GINS1*, the two unexplored genes. Silencing these genes with siRNAs in LUAD cells reduced proliferation (Figures 4G and S17B), migration (Figures 4H and S17C), and increased apoptosis (Figures 4I and S17D). Additionally, the clinical relevance of the CDSI core genes was confirmed, with these genes typically indicating worse prognosis in both published and our own datasets (Figure 4J).

### 2.7 CDSI Might be a Feature of the Proliferating T Cells

**FIGURE 5:**
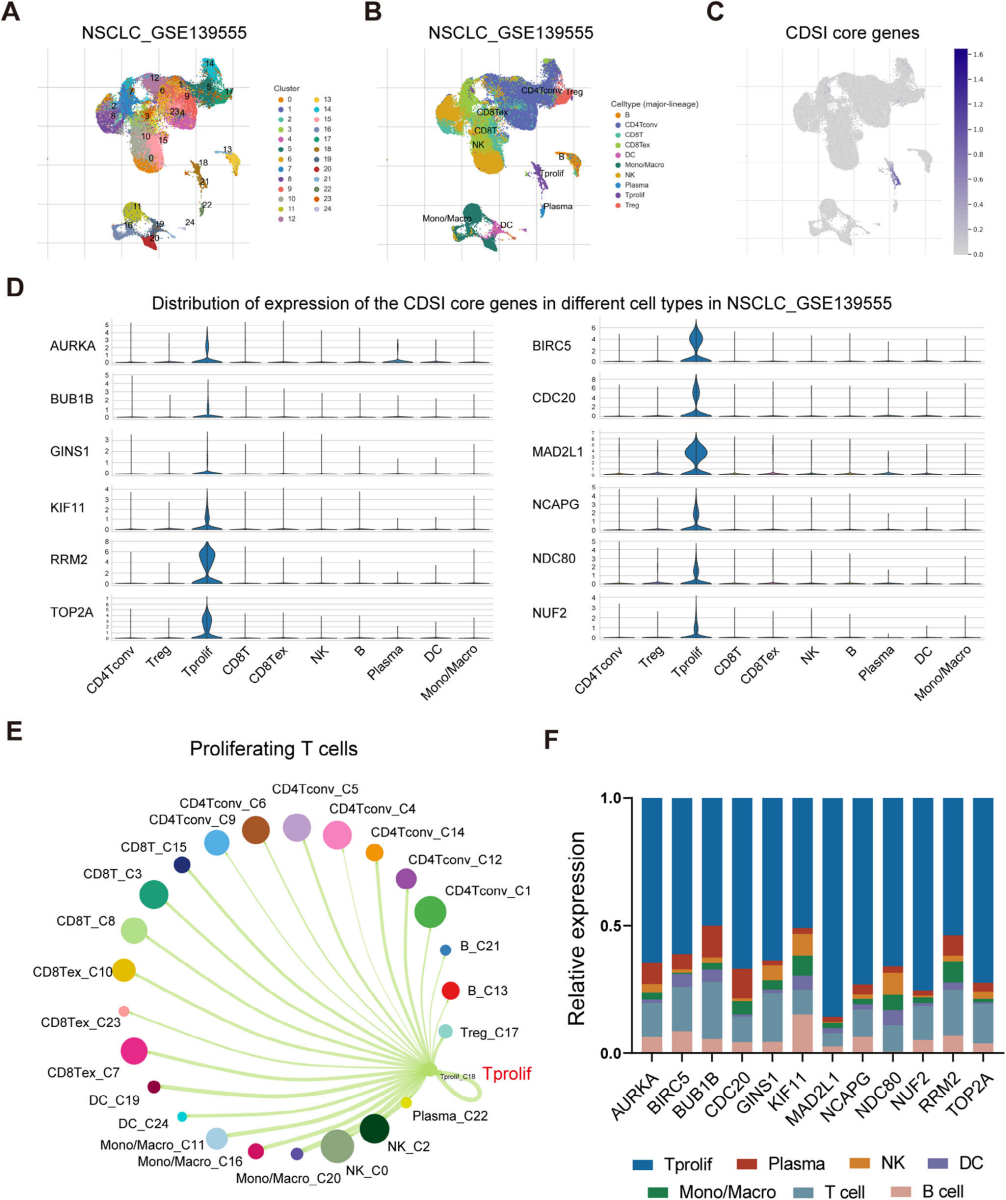
Single‐cell analysis of CDSI. (A) The cell clusters identified by UMAP in NSCLC tissues based on the GSE139555 dataset. (B) The UMAP distribution of the annotated cell types in NSCLC tissues based on the GSE139555 dataset. (C) The average expression levels of the CDSI core genes in the identified cell types in NSCLC tissues based on the GSE139555 dataset. (D) The violin plots demonstrating the expression distribution of the CDSI core genes in the identified cell types in NSCLC tissues based on the GSE139555 dataset. (E) The interactions between Tprolif cells and other cells based on the GSE139555 dataset. (F) The expression levels of the CDSI core genes in different immune cells isolated from tumor tissue in a patient with LUAD by flow cytometry.

To provide an in‐depth characterization of the TME associated with CDSI, we analyzed the single‐cell RNA sequencing (scRNA‐seq) data of T cells in non‐small cell lung cancer (NSCLC). Examination of the GSE139555 dataset identified 24 cell clusters and 10 cell types (Figure 5A,B). Our analysis revealed that the CDSI core genes were predominantly expressed in proliferating T (Tprolif) cells (Figure 5C,D). Single‐cell specific GSEA analysis indicated that in Tprolif cells, pathways such as E2F_targets, G2M_checkpoint, mitotic_spindle, and DNA_repair were significantly activated, while apoptosis, interferon_gamma response, and inflammatory_response pathways were suppressed (Figure S18). Cell‐cell interaction analysis suggested that Tprolif cells mainly interacted with natural killer cells, mono/macrophages, and dendritic cells (Figure 5E). Validation across additional NSCLC scRNA‐seq datasets confirmed higher expression of the CDSI cores genes in Tprolif cells compared with other immune, stromal, and malignant cells (Figure S19). Furthermore, flow cytometry isolation of immune cells from LUAD tumor tissue, followed by qRT‐PCR analysis, corroborated the predominant expression of the CDSI core genes in Tprolif cells (Figures 5F and S20). These results suggest that the T proliferating cell population is one of the main sources for CDSI.

### 2.8 CDSI Predicts Therapeutic Benefits in LUAD

**FIGURE 6:**
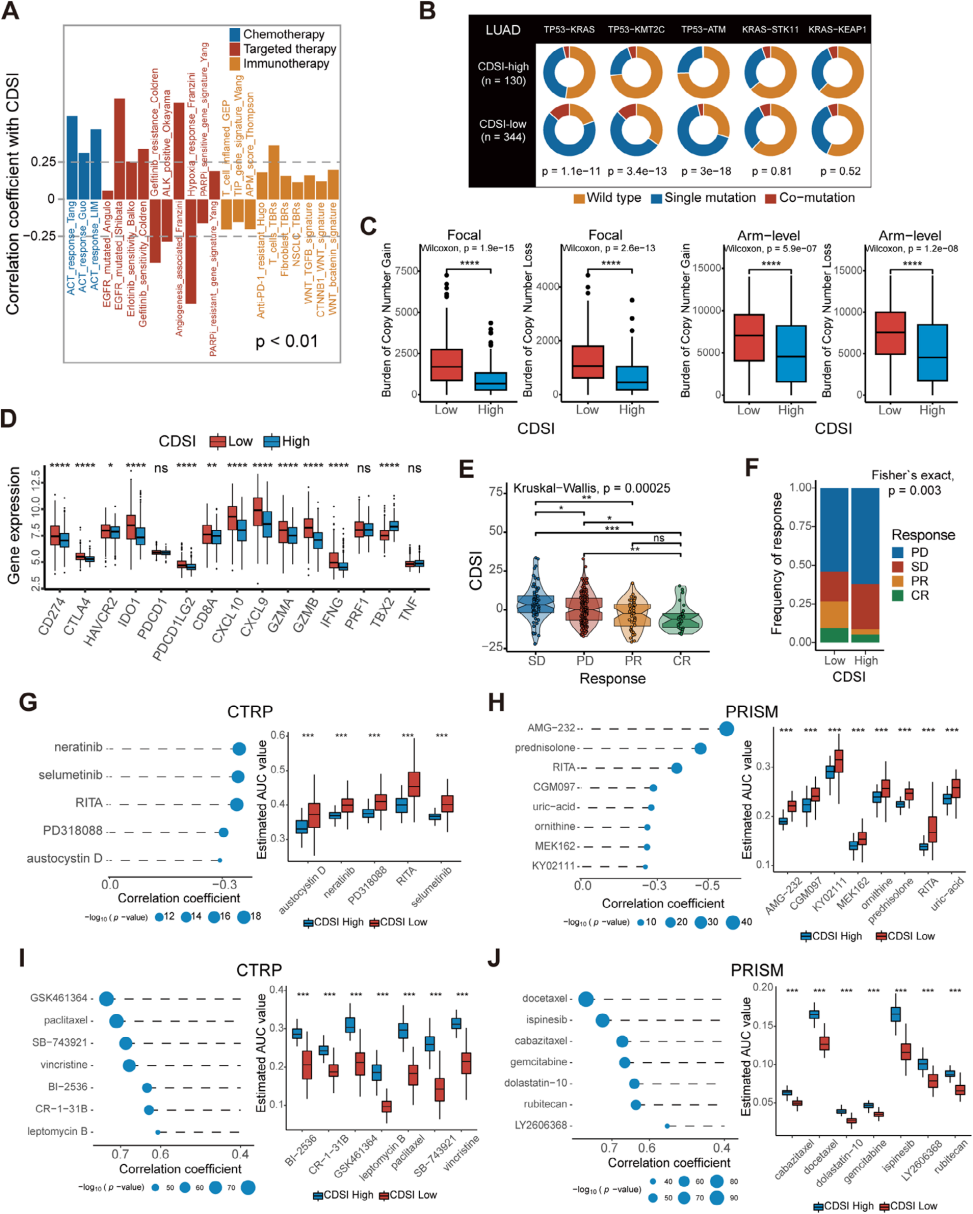
Therapeutic implications of CDSI. (A) Correlations between CDSI and therapeutic gene signatures. The *p* values were calculated using Spearman analysis. (B) Constitution of wild‐type, single mutations, and comutations among *TP53*–*KRAS*, *TP53*–*KMT2C*, *TP53*–*ATM*, *KRAS*–*STK11*, and *KRAS*–*KEAP1*. (C) Distribution of focal and broad copy number aberrations in LUADs with different CDSI phenotypes. (D) Differences in the expression of the immune checkpoint‐relevant and immune activity‐related genes between the CDSI‐high and ‐low groups. (E) Differences in CDSI between distinct clinical outcomes of anti‐PD‐L1 treatment in the IMvigor210 cohort. (F) Proportion of patients in the IMvigor210 cohort with different responses to PD‐L1 blockade immunotherapy in the CDSI‐high and ‐low groups. SD, stable disease; PD, progressive disease; CR, complete response; PR, partial response. (G–J) Results of Spearman correlation analysis and differential drug response analysis of CTRP‐derived compounds and PRISM‐derived compounds for (G and H) CDSI‐high and (I and J) ‐low tumors based on TCGA cohort. In (C–E and G–J), the upper and lower ends of the boxes represent interquartile range of values. The lines in the boxes represent median values, and black dots show outliers. *p* Values were calculated using Wilcoxon rank‐sum or Kruskal–Wallis tests. ns, not significant; *p < 0.05; **p ≤ 0.01; ***p ≤ 0.001; ****p ≤ 0.0001.

We assessed the effects of CDSI on drug response in LUAD patients. CDSI showed a positive correlation with responses to adjuvant chemotherapy, EGFR‐targeted therapy, and antiangiogenic therapy, and a negative association with immunotherapy response (Spearman |*r*| > 0.25, *p* < 0.01; Figures 6A and S21). To prove the relatedness of the CDSI with immunotherapy response, we analyzed somatic mutations from TCGA database. The mutation rates varied significantly, even among the genes that were shared between the CDSI subtypes (Figure S22A). For example, *TP53* was mutated in 62% of CDSI‐low versus 22% of CDSI‐high cases. The CDSI‐low group exhibited a more pronounced tumor mutation burden (TMB) than the CDSI‐high group (Figure S22A). The comutation rates of *TP53–KRAS*, *TP53–KMT2C*, and *TP53–ATM*, which indicated a better response to immunotherapy, were substantially higher in the CDSI‐low group, whereas those of *KRAS–STK11* and *KRAS–KEAP1*, which indicate an inferior immunotherapy response, were similar between the groups (Figure 6B) [31]. Copy number (CN) aberrations were also assessed, with the CDSI‐low group exhibiting higher focal and arm‐level gains/losses (Wilcoxon test, *p* < 0.001; Figure 6C). Other measures of DNA damage [23], including aneuploidy score, homologous recombination defects (HRDs), and intratumor heterogeneity (ITH), were substantially and consistently higher in the CDSI‐low group (Figure S22B–D). The CDSI‐low group also had a higher neoantigen burden (Figure S22E,F) [23]. Moreover, the Wilcoxon test showed that most immune‐checkpoint‐relevant and immune‐activity‐related genes were overexpressed in the CDSI‐low group (*p* < 0.05; Figure 6D) [32, 33]. These analyses indicate that patients with low CDSI may benefit from immunotherapy. In the IMvigor210 cohort (Table S17), patients with better responses to immune checkpoint inhibitors had lower CDSI (Kruskal–Wallis, *p* = 0.00025; Figure 6E), and the CDSI‐low group had more patients with clinical benefits than the CDSI‐high group (Fisher's exact test, *p* = 0.003; Figure 6F). Consistently, the CDSI was lower in the “immune inflamed” type than in the “immune excluded” and “immune desert” groups (Figure S22G). Higher TMB and neoantigen burden were observed in the CDSI‐low group (Figure S22H,I). These results may explain the greater benefit of ICI treatment for the CDSI‐low group.

We further applied the Cancer Therapeutics Response Portal (CTRPv2) and PRISM data to delineate drug sensitivity associated with the CDSI subtype in TCGA cohort [34, 35]. We identified 12 compounds that may benefit CDSI‐high patients and 14 that could benefit CDSI‐low patients (Figure 6G–J). CDSI‐high patients might respond to ERK/MAPK inhibitors such selumetinib and PD318088, and MDM2–p53 pathways inhibitors like AMG‐232 and RITA. In contrast, CDSI‐low tumors could be sensitive to mitosis pathways inhibitors including GSK461364 and SB−743921.

### 2.9 Diagnostic, Prognostic, and Therapeutic Values of CDSI Across 32 Cancer Types

**FIGURE 7:**
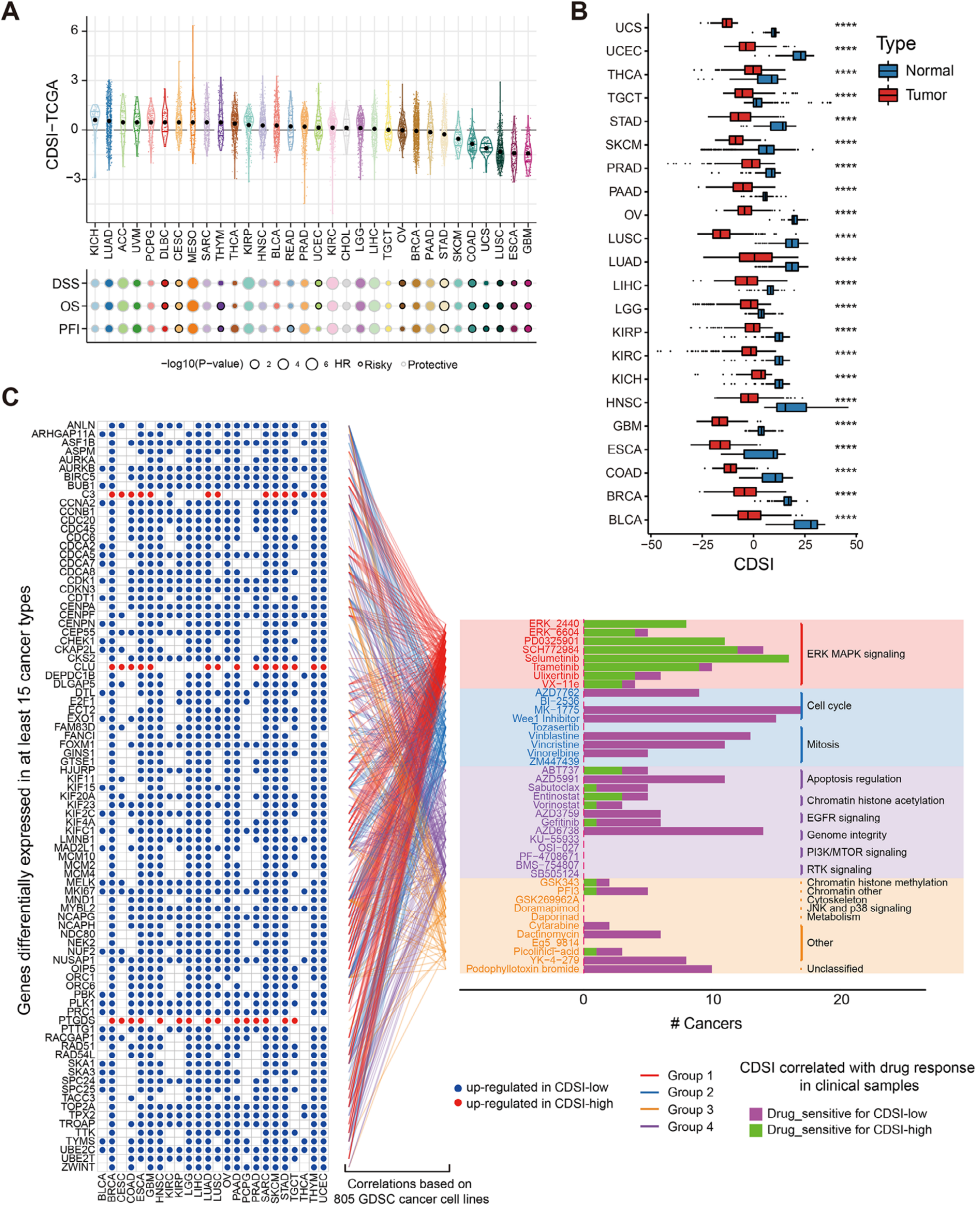
Clinical utility of CDSI in various cancers. (A) Distribution of the CDSI among various TCGA cancer types and its prognostic implications. The *p* values were calculated using the log‐rank test. DSS, disease‐specific survival; PFI, progression free interval. (B) Difference in CDSI between tumor and normal samples across multiple cancer types. The upper and lower ends of the boxes represent the interquartile range of values, the lines in the boxes represent median values, and black dots indicate outliers. *p* Values were tested using the Wilcoxon rank‐sum test. ****p ≤ 0.0001. (C) Identification of potential drugs for various cancers with different CDSI levels via integrated analysis. The left panel shows the genes differentially expressed between the CDSI‐high and ‐low tumors (CDSI‐DEGs). Blue indicates upregulation in the CDSI‐low tumors, while red indicates upregulation in the CDSI‐high tumors. The lines connecting these genes (left) to the drugs (right) represent their associations as determined via Spearman analysis using CCLs data. The barplots in the right panel indicate the number of cancer types with positive (drug‐sensitive for CDSI‐low, magenta) and negative (drug‐sensitive for CDSI‐high, green) correlation between CDSI and drug response in clinical samples (Spearman's rank correlation). These drugs are categorized by their targeted pathways. To highlight key target pathways, these drugs are also classified into four groups based on the number of drugs targeting the same pathway.

Pan‐cancer analysis unveiled substantial variations in CDSI across different tumor types and histological subtypes (Figure 7A, upper panel). Univariate Cox analysis using OS, disease‐specific survival, and progression‐free interval demonstrated that, similar to LUAD, a higher CDSI predicted better prognosis in most cancer types (Figure 7A, lower panel). Comparing CDSI between tumor and normal samples, with exclusion of cancer types having ≤20 normal samples, exhibited revealed significantly higher CDSI in all cancers compared with normal samples (Figure 7B), implying that the CDSI may serve as a diagnostic biomarker for cancer.

To characterize the therapeutic implications of CDSI, we identified 112 DEGs between the CDSI‐high and ‐low groups in ≥15 cancer types (refer to as CDSI‐DEGs). The expression of these genes in each of the 805 cell lines from the Genomics of Drug Sensitivity in Cancer (GDSC2) was correlated with the half‐maximal inhibitory concentration (IC_50_) obtained in the same cell line [36]. Compounds with a significant relationship (Spearman *r* ≥ 0.25, *p* < 0.05) with at least four CDSI‐DEGs were regarded as candidates, yielding 41 potential drugs (Table S19). These drugs could be categorized according to their target pathways. Identifying more drugs targeting the same pathway indicates that the pathway may better determine the therapeutic outcomes of the two groups. Correlation analysis between patient‐derived IC50 values of candidate drugs and CDSI for each cancer type defined sensitivity in CDSI‐high and CDSI‐low tumors as Spearman *r* < −0.3 and >0.3, respectively (*p* < 0.05). The results closely mirrored our observations from the LUAD datasets. Patients with high CDSI may benefit from ERK/MAPK signaling pathway inhibitors like selumetinib and PD0325901 (sensitive in 16 and 11 cancer types, respectively), whereas CDSI‐low tumors could be controlled by cell cycle and mitosis inhibitors such as MK‐1775 and vinblastine (sensitive in 17 and 13 cancer types, respectively) (Figure 7C).

## 3 Discussion

Both RCD and CS are established cancer hallmarks with profound effects on tumorigenesis [13]. For the first time, we conducted a systematic investigation of the expression and interacting patterns of 21 CDS features in LUAD, identified three distinct CDS regulation patterns, developed an individualized prediction model termed CDSI, and confirmed its potency as an indicator of patient prognosis and treatment response across various datasets.

Typically, RCD and CS showed a lower and higher expression in tumors than in normal samples, respectively. Considerable crosstalk exists among the CDS features as suggested by the identification of four CDS modules. The concept of PANoptosis, which implies intricate cross‐regulation among pyroptosis, apoptosis, and necroptosis during pathogenic infection and tissue development [37], may also be initiated in LUAD. Our results indicate that these cell death pathways were tightly connected in module‐1.

Our study confirms the presence of three distinct CDS regulation patterns in LUAD and their crucial role in shaping the TME. Based on these CDS features, we established three LUAD subtypes, each with unique tumor intrinsic and TME features. CDS‐A is characterized by low module‐2 activity, moderate module‐1 activity, moderate TME infiltration, and favorable prognosis; CDS‐B by high module‐1 activity, moderate module‐2 activity, high TME infiltration, and poor prognosis; CDS‐C by high module‐2 activity, low module‐1 activity, high cell cycle signaling, and a desert‐like TME with the lowest immune and stromal scores. Module‐1, comprising typical ICD modes, correlates positively with nearly all TME‐infiltrating cells, including immunosuppressive cells such as CAFs, regulatory T cells, and myeloid‐derived suppressor cells [38]. This module may induce an immune‐excluded phenotype in CDS‐B, negatively impacting patient prognosis. Module‐2, based on CS features, is associated with cell cycle signaling pathways that promote tumor growth, with high activity in CDS‐C potentially worsening patient outcomes. CDS‐A exhibits increased angiogenesis, innate immune infiltration, and a unique fibroblast phenotype characterized by increased universal fibroblasts and decreased CAFs. Universal fibroblasts, found across different tissues, mainly in normal tissues, can generate specialized fibroblasts such as CAFs [39]. While module‐1 promotes fibroblast accumulation in the TME, module‐2 drives their differentiation into activated fibroblasts, creating a more tumor‐permissive microenvironment. Our speculations are supported by the enrichment of pan‐iCAF and pan‐iCAF‐2 subtypes [40], representing universal fibroblasts [39], in CDS‐A, and the pan‐myCAF, pan‐dCAF, and pan‐pCAF subtypes, associated with TME‐favoring ECM remodeling, in CDS‐B.

The CDSI, obtained by principal component analysis (PCA), has been shown to predict individual tumor CDS regulation patterns. Subsequent analyses with ≥200 functional gene signatures indicated that the CDSI was negatively associated with adaptive immunity, CAFs, cell cycle, and cancer stemness but positively associated with innate immunity, angiogenesis, and universal fibroblasts. Patients with LUAD who presented a high CDSI demonstrated a significant survival benefit over those with low CDSI. Validation across multiple cohorts, including those of Chinese descents, confirmed the CDSI as an independent predictor of better OS for LUAD. This unique population, characterized by distinct genetic and environmental factors, differs from the LuMMD cohort where the model was developed. Moreover, these independent datasets were generated using different techniques, including Affymetrix microarray, Illumina beadchip, and Illumina RNA‐seq. These results suggest that our model accurately predicts patient prognosis across diverse populations and profiling platforms.

To explain the survival discrepancies, we conducted molecular characterization. Unlike the CDSI‐high group, which primarily consisted of CDS‐A tumors, the CDSI‐low LUADs, mainly CDS‐B and ‐C, were marked by a high proliferation rate and immune‐suppressive TME. CRISPR‐screened data enabled us to identify 12 CDSI core genes that were significantly upregulated in CDSI‐low LUAD tumors, forming a cohesive interaction network. We confirmed the association of the genes with CDSI using LUAD cell lines. In vitro studies demonstrated that knockdown of *MAD2L1* and *GINS1*, two of the CDSI core genes, suppressed proliferation and migration while promoting apoptosis, consistent with the functions of the other CDSI core genes. Our results consistently showed that these core genes were indicative of poor prognosis.

Subsequent analysis suggested that the expression of the CDSI core genes was enriched in Tprolif cells across various single‐cell datasets. This is consistent with recent studies showing an enrichment of genes related to cell death processes such as cuproptosis and anoikis in Tprolif cells [41, 42]. Moreover, previous research has documented the critical role of Tprolif cells in the early stages of CD8+ T cell dysfunction [43], characterized by high expression of immune exhaustion marker genes like *LAG3*, *HAVCR2*, *PDCD1* [43, 44, 45]. The presence of these immunosuppressive genes in Tprolif cells may help elucidate the immune microenvironment status of the CDSI‐low tumors and the unfavorable prognosis of associated patients. Our findings provide a partial explanation for the survival advantage observed in CDSI‐high tumors.

The CDSI has emerged as a promising indicator for tailoring treatment strategies in LUAD. It shows a positive correlation with responses to adjuvant chemotherapy, EGFR‐targeted therapy, and antiangiogenic therapy, while negatively correlating with immunotherapy response. Notably, patients responding to anti‐PD‐L1 immunotherapy exhibited a substantially lower CDSI. In line with this observation, genomic features predictive of immunotherapy benefits were significantly elevated in the CDSI‐low group, including CD274 (PD‐L1) expression, TMB, CN alterations, aneuploidy score, HRDs, ITH, and neoantigen burden. Gene comutations associated with improved immunotherapy response were also enriched in the CDSI‐low group. Furthermore, the Tprolif cells, likely targeted by CDSI based on single‐cell analysis, were enriched in dysfunctional T cells, which are associated with improved tumor reactivity [43].

In addition, several compounds that might benefit the CDSI subgroups were screened from multiple drug databases. High CDSI patients may benefit from drugs targeting ERK/MAPK (e.g., selumetinib, PD0325901) and MDM2–p53 pathways (e.g., AMG‐232, Nutlin‐3a), whereas the CDSI‐low tumors could be controlled by drugs targeting cell cycle and mitosis pathways (e.g., Paclitaxel, MK‐1775). Overall, the CDSI predictive model is advantageous in determining therapies for both CDSI‐low and ‐high patients with LUAD, and should be cost effective in clinical application.

Our analysis extended beyond LUAD to include a broader spectrum of cancer types, revealing that normal samples across all cancers had significantly higher CDSI than tumor samples, demonstrating the diagnostic value of the CDSI. In most cancer types, a higher CDSI predicted better survival. We also uncovered the therapeutic value and potential mechanisms related to the CDSI in different cancers. Consistent with LUAD, the CDSI correlated with resistance to cell cycle and mitosis pathway‐targeting drugs and sensitivity to ERK/MAPK pathway inhibitors. These findings validate the robustness and broad applicability of our analysis.

Our study, however, had limitations. The molecular mechanisms underlying the CDS modes have not been fully elucidated, which may limit the precision of the gene signatures used to quantify their activities. Additionally, our reliance on mRNA‐level quantifications may introduce inaccuracies, as the CDS processes are protein dependent. Retrospective patient recruitment could introduce selection bias, and a critical cutoff value for CDSI in assessing prognosis and treatment responses across different datasets has yet to be determined. Further experimental and clinical validations are necessary for accelerating the clinical translation of our findings.

Nonetheless, our CDSI model is a clinically valuable tool for identifying patients with unique CDS regulation patterns and predicting patient survival and therapeutic benefits in LUAD and other cancer types. This study provides novel insights into CDS regulation in relation to the TME, potentially enhancing precision therapy strategies for cancer patients.

## 4 Materials and Methods

### 4.1 Datasets and Source

The LuMMD was downloaded from ArrayExpress using E‐MTAB‐6699. Raw data of 1621 patient‐derived samples from 12 independent Gene Expression Omnibus (GEO) studies were preprocessed and normalized using an integrated bioinformatics pipeline (Table S2) [46]. Author‐processed data of GSE41271 and E‐MTAB‐923 were directly retrieved from GEO and ArrayExpress. RNA‐seq data of the LUAD‐Singapore cohort were obtained from the Singapore Oncology Data Portal and normalized to transcripts per million (TPM) [47]. TCGA and Genotype‐Tissue Expression RNA‐seq data (TPM normalized) were downloaded from the UCSC Xena portal in September 1^st^, 2021(Table S20). Clinical data corresponding to these datasets were retrieved and manually curated when available. Expression and clinical data of the immunotherapeutic cohort, IMvigor210, were obtained from the “IMvigor210” package (version 1.0.0) in R [48]. Human cancer cell line (CCL) expression data were obtained from the Broad Institute CCLE project and GDSC database [36, 49].

### 4.2 Collection and Quantification of Gene Signatures

The gene signatures characterizing CDS features were primarily collected from REACTOME, Gene Ontology (GO), and FerrDb [50]. For less‐studied features, signatures were generated by curating published studies (Table S1). The activities of 21 CDS features were calculated using single‐sample GSEA (ssGSEA) using the GSVA package (version 1.42.0) in R [51]. To comprehensively characterize the TME, we collected 256 gene signatures from 40 published studies (Table S21). These signatures were quantified via ssGSEA, GSVA, or methods provided by the original authors. Detailed descriptions are available in Supporting Information.

### 4.3 Consensus Clustering for CDS and Genomic Subtypes

We clustered LUADs based on the ssGSEA scores of 21 CDS features in the LuMMD cohort. Analysis was performed using the ConsensusClusterPlus package (version 1.58.0) in R with these parameters: [52] clusterAlg = “km,” innerLinkage = “ward.D,” finalLinkage = “ward.D,” distance = “Euclidean,” reps = 1000, pItem = 0.8, and pFeature = 1. The genomic subtypes were identified by consensus clustering of the CDS‐associated DEGs using the same parameters, except clusterAlg = “pam.”

### 4.4 Identification of DEGs Between CDS Subtypes

CDS‐associated DEGs were determined using the limma package (version 3.50.3) in R [53], with the cutoff criteria for significance set to a false discovery rate (FDR) < 0.05 and absolute log fold‐change (|log_2_|FC) > 0.8.

### 4.5 Supervised Clustering of TCGA LUADs

To predict CDS subtypes in TCGA cohort, a supervised analysis was performed [54]. The top 100 upregulated genes (FDR < 0.25) in each CDS subtype were defined as marker genes, and hierarchical agglomerative clustering (using Pearson's distance and Ward's linkage) was applied based on the marker genes. The number of clusters was set as three.

### 4.6 Functional Enrichment Analysis

To investigate the biological processes underlying each CDS module, GSVA was performed using the GSVA R package and the “Hallmark” gene sets of the MSigDB database. We compared the GSVA scores for the module using a generalized linear model [53]. To explore the difference in biological functions between CDS subtypes, first, we conducted GSEA using subtype‐specific genes, which were determined by calculating the expression fold changes of one subtype versus all other subtypes using limma. The gene sets of “c2.cp.v7·4” and “c5.go.v7·4” downloaded from MSigDB were used for GSEA and ranked according to normalized enrichment scores (NES) [55]. The top 10 representative gene sets with the largest NES were selected for each subtype and used for heatmap visualization.

### 4.7 Generation of CDSI

DEGs that were positively and negatively correlated with the genomic clusters were defined as CDS gene signatures A and B, respectively. These signatures underwent dimension reduction using the Boruta algorithm (Boruta R package Version 7.0.0) [56]. PCA was conducted to determine the signature score using principal component 1. We used a gene expression grade index (GGI)‐like method to define the CDSI of each patient: [57]

(1) $$CDSI=\sum PC1_{B}-\;\sum PC1_{A}$$

### 4.8 Identification of the CDSI Core Genes and Functional Similarity Analysis

Data from genome‐wide CRISPR screening of LUAD cells were downloaded from the DepMap portal (https://depmap.org/portal/download/). Dependency scores were calculated using the CERES algorithm [27]; genes with a CERES score less than one across 75% of LUAD cell lines (*n* = 51) were identified as essential. The essential genes that were simultaneously differentially expressed (|log2FC| ≥ 1 and FDR < 0.05) between tumor and normal samples, as well as the CDSI‐high and ‐low samples in both the LuMMD and TCGA cohorts, were defined as CDSI core genes.

To identify hub genes that play key roles for the interactome to exert functions, we ranked the core genes based on average functional similarities between each gene and its interaction partners. Functional similarity was defined as the geometric mean of semantic similarities in the molecular function and cellular component aspects of GO, which were measured using the GOSemSim R package and the Wang method [30]. A cutoff value of 0.75 was chosen to define hub genes.

### 4.9 Cell Culture and RNA Interference

The human lung CCLs NCI‐H1395, NCI‐H2126, HCC‐4006, NCI‐H358, NCI‐H1650, and NCI‐H2009 cells (ATCC, USA) were cultured in RPMI‐1640 medium (Corning, Corning, NY, USA) supplemented with 10% fetal bovine serum (FBS) (Gibco, Waltham, MA, USA) and 1% penicillin–streptomycin (Invitrogen, Carlsbad, CA, USA) to prevent microbial contamination. Cells were maintained in a humidified incubator at 37°C with 5% CO_2_. A549 cells were selected for *MAD2L1* and *GINS1* knockdown. siRNAs targeting these genes (three for each gene) were obtained from Genechem Company (Daejeon, South Korea). Transfections were performed using Lipofectamine 3000 (Invitrogen) according to the manufacturer's protocol, with the siRNAs diluted in the cell culture medium.

### 4.10 In Vitro Validation of the CDSI Core Genes

Gene expression levels were quantified using qRT‐PCR and protein expression was analyzed by western blotting. A suite of assays was conducted to explore the biological functions of the genes under investigation. Cell viability was assessed using the cell counting kit‐8 (CCK‐8) assay. Transwell assays were performed to examine cell migration and invasion capabilities. Flow cytometry was employed to investigate cell apoptosis. Detailed experimental methods are provided in the Supporting Information.

### 4.11 Prognostic Validation of the CDSI Core Genes

Seventy‐six patients diagnosed with LUAD at the National Cancer Center were enrolled in this study (NCC‐LUAD). We collected specimens through surgical resection following stringent standard operating procedures, with the samples then archived as formalin‐fixed paraffin‐embedded tissue blocks. Patient characteristics are detailed in Table S22. Ethical approval for this study was obtained from the Ethics Committee of the Cancer Hospital, Chinese Academy of Medical Sciences (approval number 20/451‐2647). Gene expression was quantified using qRT‐PCR, with the full experimental protocols detailed in the Supporting Information.

### 4.12 Single‐Cell Analysis of the CDSI Core Genes

Expression profiles of the CDSI core genes across various cell types in TME were analyzed using the Tumor Immune Single‐cell Hub 2 (TISCH2) [58]. We utilized seven NSCLC scRNA‐seq datasets (GSE139555, GSE153935, GSE148071, GSE162498, GSE99254, GSE176021_aPD1, GSE151537) and applied a uniform processing workflow that encompassed quality control, batch effect correction, cell clustering, and cell‐type annotation.

To validate our single‐cell analysis, we prepared single‐cell suspensions from LUAD tumor tissues through enzymatic digestion. Surface staining was performed on ice for 30 min using a panel of antibodies targeting specific surface antigens in a flow cytometry buffer composed of PBS with 1% FBS. The antibodies used included CD45 (#982316), CD20 (#980206), CD3 (#981012), CD11b (#982606), CD14 (#982506), CD56 (#985902), CD38 (#980308), CD138 (#390207), and Ki67 (#350503), all obtained from BioLegend, and were used following the manufacturer's guidelines. Sorted cells were collected for total RNA extraction, reverse transcribed into cDNA, and subjected to qRT‐PCR to evaluate the expression levels of the CDSI core genes.

### 4.13 Somatic Mutation and CN Variation Analyses

Somatic mutation data and CN segment data of LUAD were downloaded via TCGAbiolinks (version 2.22.4) and analyzed using the maftools R package (version 2.10.05) and GISTIC 2.0, respectively [59, 60, 61]. The burden of CN loss or gain was calculated as the total number of genes with CN changes at the focal and arm levels.

### 4.14 Prediction of Therapeutic Response

The drug sensitivity profiles of hundreds of CCLs, measured as either the area under the (dose–response) curve (AUC) values or IC_50_, were obtained from the CTRPv2, PRISM Repurposing dataset, and GDSC [34, 35, 36]. The estimated AUC or IC_50_ of each compound in each clinical sample was predicted using the oncoPredict package (version 0.2) in R [62]. Lower AUC and IC_50_ values indicate increased sensitivity to treatment. Differential and correlation analyses were performed to identify potential therapeutic agents for selected patients with LUAD [63]. Additional details are shown in the Supporting Information.

### 4.15 Statistical Analysis

All statistical analyses were conducted using R (version 4.1.1). For continuous variables, we employed unpaired Student *t* and Wilcoxon rank‐sum tests for comparisons between two groups, and the Kruskal–Wallis test for comparisons among more than two groups. Categorical variables were assessed using Fisher's exact test. Correlations between variables were calculated using the Spearman method, unless otherwise indicated. The MaxStat R package (version 0.7‐25) was utilized to dichotomize patients based on CDSI or gene expression levels, identifying cut points that maximized the rank statistic and minimizing computational batch effects. Survival analyses were performed using the KM method with the Survminer package (version 0.4.9) in R, with significant differences identified using a log‐rank test. The HR was estimated using a Cox regression model with the Survival R package (version 3.3‐1). A multivariate Cox regression model was used to determine independent prognostic factors. The net reclassification index was estimated using the nricens R package (version 1.6). Patients with a survival time of less than 30 days were excluded from survival analyses. For all statistical analyses, statistical significance was set at two‐tailed *p* < 0.05.

## Author Contributions

*Conception and design*: Pan Wang, Chaoqi Zhang, and Jie He. *Acquisition and analysis of data*: Pan Wang, Chaoqi Zhang, and Zhihong Zhao. *Collection of clinical samples and in vitro experiments*: Chaoqi Zhang and Peng Wu. *Writing, review, and/or revision of the manuscript*: Pan Wang, Chaoqi Zhang, Peng Wu, Zhihong Zhao, Nan Sun, and Qi Xue. S*tudy supervision*: Jie He and Shugeng Gao. All the authors reviewed and approved the final manuscript.

## Ethics Statement

For the clinical samples and associated patient data from the NCC‐LUAD cohort, ethical approval obtained from the Ethics Committee of the Cancer Hospital, Chinese Academy of Medical Sciences (approval number 20/451‐2647). The remaining patient data utilized in this study were obtained from publicly accessible datasets, where patient consent had been previously secured.

## Conflicts of Interest

The authors declare no conflicts of interest.

## Supporting information



Supporting Information

Supporting Information

## Data Availability

The microarray or RNA‐sequencing data and associated clinical information of cancer patients as well as the expression and drug sensitivity data of human CCLs are described under “Datasets and source” in the Supporting Information. The resources, tools, and codes used in our analyses are described in detail in Supporting Information.
