# Supplementary material for: Cell Death and Senescence‐Based Molecular Classification and an Individualized Prediction Model for Lung Adenocarcinoma
Source: MedComm (2020). 2025 May 29;6(6):e70237. doi: 10.1002/mco2.70237 (PMC12122187; doi:10.1002/mco2.70237)
Supplement: Supplementary file 1 — Supporting Information [file MCO2-6-e70237-s001.docx]

**Cell death and senescence-based molecular classification and an individualized prediction model for lung adenocarcinoma**

Pan Wang^1^, Chaoqi Zhang^1^, Peng Wu^1^, Zhihong Zhao^1^, Nan Sun^1^, Qi Xue^1^, Shugeng Gao^1,*^, and Jie He^1,*^

^1^Department of Thoracic Surgery, National Cancer Center/National Clinical Research Center for Cancer/Cancer Hospital, Chinese Academy of Medical Sciences and Peking Union Medical College, Beijing 100021, China

***Corresponding authors:**

Shugeng Gao ([gaoshugeng@cicams.ac.cn](mailto:gaoshugeng@cicams.ac.cn))

Jie He ([prof.jiehe@gmail.com](mailto:prof.jiehe@gmail.com))

Address: Department of Thoracic Surgery, National Cancer Center/National Clinical Research Center for Cancer/Cancer Hospital, Chinese Academy of Medical Sciences and Peking Union Medical College, No. 17 Panjiayuannanli, Beijing, 100021, People’s Republic of China. Phone: 86-10-87788863. Fax: 86-10-67713359.

Jie He will handle correspondence at all stages of refereeing and publication, also post-publication.

# SUPPLEMENTARY MATERIALS AND METHODS

## Detailed descriptions of the gene signatures used in this study

To comprehensively characterize the tumor microenvironment (TME) of lung adenocarcinoma (LUAD) and explore the biological significance of LUAD mediated by cell death and senescence (CDS), we collected a total of 256 gene signatures from 40 published studies (Table S4). Specifically, a total of 28 pancancer metagenes for characterizing immune cell subpopulations were defined by Charoentong et al.,^1^ and they were quantified using single-sample gene set enrichment analysis (ssGSEA).^2^ Tumor stroma is typically composed of extracellular matrix (ECM) and specialized connective tissue cells, including fibroblasts, endothelial cells, and mesenchymal stromal cells. For endothelial cells, two signatures, which differentiate vascular lineage from lymphatic lineage, were selected from the single-cell analyses of endothelial cells of the human lung tissues.^3^ Another three sets of marker genes that represent normal endothelial cells, tumor endothelial cells, and lymphatic endothelial cells were obtained from the single-cell study of stromal cells in the lung tumor microenvironment.^4^ In addition, we collected two gene signatures identified using integrated analysis of bulk RNA expression, which underly angiogenesis in non-small cell lung cancer (NSCLC) and pancancer.^5, 6^ In terms of fibroblasts, a recent study indicated the existence of universal, specialized, and disease-specific subsets of fibroblasts.^7^ The universal fibroblasts were found across different tissues, mainly in normal tissues, and served as a reservoir that could yield specialized fibroblasts, including cancer-associated fibroblasts (CAFs). The authors showed that activation of human fibroblasts might be associated with loss of universal fibroblast gene expression. Thus we collected these two signatures as “universal fibroblast” and “fibroblast activation”.^7^ CAFs have been widely described as key players in tumor stroma that promote tumorigenesis through multiple mechanisms. Here, we collected a CAF gene signature obtained by comparing the gene expression between CAFs and matched normal fibroblasts from 15 patients with resected NSCLCs.^8^ To reveal the heterogeneity of CAFs, gene signatures for six CAF subtypes, identified across multiple cancer types, were retrieved from a recent study.^9^ The activated stromal gene signature that captures the activated inflammatory stromal response was also obtained.^10^ To describe ECM, we utilized two sets of ECM genes that were upregulated and downregulated in cancer (C-ECM).^11^ Since the C-ECM transcriptional program dysregulation is correlated with the activation of TGF-β signaling in CAFs, we named these signatures as “CAF ECM up” and “CAF ECM down”. Signatures of ECM glycoproteins, ECM collagens, and ECM proteoglycans were downloaded from the MatrisomeDB database.^12^ These gene signatures of tumor stroma were also quantified using ssGSEA.

In this study, the overall infiltration of immune and stromal cells was calculated using the ESTIMATE algorithm.^13^ To validate the TME characteristics, we included 29 manually curated functional gene expression signatures (Fges29) that represent the major functional components and immune, stromal, and other cellular populations of the tumor.^14^ In addition, we collected another reference immunome compendium consisting of 28 gene signatures (Bindea), representing different immune cell types infiltrating tumors.^15^ Moreover, a set of characteristic genes, whose expressions uniquely define several major types of immune cells derived from the ImmGen database, were also incorporated (Davoli).^16, 17^ The above gene signatures were quantified using ssGSEA. Furthermore, we took advantage of several algorithms, which are integrated in the IOBR R package,^18^ to independently quantify the cellular components in the TME, including CIBERSORT,^19^ EPIC,^20^ MCP-Counter,^21^ quanTIseq,^22^ TIMER,^23^ and xCell.^24^

To investigate the TME characteristics of the CDSI phenotypes, we used gene sets curated by Mariathasan and colleagues,^25^ including CD8 T-effector signature; immune checkpoint signature; antigen processing machinery; mismatch repair; nucleotide excision repair; DNA damage repair; DNA replication; homologous recombination; pan-fibroblast TGFb response signature (Pan-F-TBRS); epithelial-mesenchymal transition markers, including EMT1, EMT2 and EMT3; FGFR3-related genes; DNA replication dependent histones (KEGG discovered histones); angiogenesis signature; Fanconi anemia pathway that is required for the efficient repair of damaged DNA; WNT target; cell cycle genes; and cell cycle regulators. We also collected several therapeutic signatures, including gene signatures predicting adjuvant chemotherapy responses,^26-28^ targeted therapy-associated gene signatures,^29-35^ gene signatures that are correlated with immunotherapy responses,^36-38^ and oncogenic pathways that could shape a non-inflamed TME.^39-43^ The above gene signatures were quantified using gene set variation analysis (GSVA).^2^

## Prediction of therapeutic response

Drug sensitivity profiles of hundreds of cancer cell lines were obtained from the Cancer Therapeutics Response Portal (CTRPv2), PRISM Repurposing dataset, and the GDSC database.^44-46^ As measurement of drug sensitivity, area under the dose-response curve (AUC) values or half-maximal inhibitory concentration (IC50) was utilized. Compounds with more than 20% of missing data were excluded. Cell lines derived from hematopoietic and lymphoid tissue were also excluded from CTRP. K-nearest neighbor (k-NN) imputation was applied to impute the missing values. Then, the calcPhenotype function of the oncoPredict R package was used to estimate the drug sensitivity of clinical samples through ridge regression using default parameters.^47^

## Quantitative reverse-transcription PCR (qRT-PCR) for cell lines and tumor tissues

Total RNA from cell lines was extracted using the RNA-Quick Purification Kit (ES-RN001) according to the manufacturer's protocol. For FFPE samples, RNA was isolated using the RNeasy FFPE Kit (QIAGEN, 73504). The PrimeScript™ RT Reagent Kit (Tiangen, KR116) was used to reverse transcribe RNA samples into cDNA, which was then analyzed using the QuantiNova SYBR Green PCR Kit (Qiagen, 208054) on an Agilent Mx3005P Real-Time PCR System. Each sample was assayed in triplicate, with GAPDH as the internal reference gene. Gene expression levels were quantified using the comparative CT (2^−ΔΔCt) method for relative quantification.

Primers used for RT-qPCR are listed as following:

BIRC5 (Forward primer: CGAACCCCAGACCTGTTTGT, Reverse primer: GGCTGGAGTGCATTTTCTGC)

KIF11 (Forward primer: GATGGACGTAAGGCAGCTCA, Reverse primer: TGTGGTGTCGTACCTGTTGG)

RRM2 (Forward primer: TTGGTGGAGCGATTTAGCCA, Reverse primer: GCCTCTTTGTCCCCAATCCA)

MAD2L1(Forward primer: ACGGTGACATTTCTGCCACT, Reverse primer: TGGTCCCGACTCTTCCCATT)

AURKA (Forward primer: ATCGTGCAGGGGGAGAAATC, Reverse primer: GAGGGCAGCAGTCAATGGTA)

CDC20(Forward primer: GGCTATGGCGCTGTTTTGAG, Reverse primer: TGGTGGATGAGGCTGCTTTT)

GINS1 (Forward primer: AGCAAAGTCAGGTGGACGAA, Reverse primer: ACAGTGCAGCGTCGATTTCT)

BUB1B (Forward primer: GGATGGGTCCTTCTGGAAACT, Reverse primer: CACTGAAAGAGCAAAGCCCC)

NCAPG (Forward primer: AGCAGATGTCCATTTCAACAGG, Reverse primer: GTCCACAGCATCCCAAGCATA)

TOP2A (Forward primer: GACAGGTGGTCGAAATGGCT, Reverse primer: CCATCTCACCAGCTCTTCCC)

NUF2 (Forward primer: AACAGTTAAACGCCGCACAC, Reverse primer: TTCCCTCTTGCAGCACTATCG)

NDC80 (Forward primer: ATCAAGGACCCGAGACCACT, Reverse primer: CGTATGAGGGGCACAGGAAG)

GAPDH (Forward primer: GGACCTGACCTGCCGTCTAG, Reverse primer: GGACCTGACCTGCCGTCTAG)

## Western Blot and Antibodies

Cell samples were washed with Phosphate buffer saline (PBS, #D8537; Sigma-Aldrich) for three times, and cell proteins were extracted using RIPA lysis reagent (P0013B, Beyotime, China) supplemented with protease and phosphatase inhibitor (Thermo Fisher Scientific, No. 78442). Then, the protein concentration was quantified with a BCA protein assay kit (Thermo Fisher Scientific, No. 23227) according to the manufacturer's protocol. Equal amounts of proteins were separated by 10% SDS-PAGE (Vazyme, China) and subsequently transferred to proteins to polyvinylidene fluoride (PVDF) membranes (Merck, Burlington, MA, USA). The primary antibodies for Western blotting used in this study are listed as follows: NCAPG (1:5000, #24563-1-AP, proteintech, USA), MAD2L1 (1:1000, #10337-1-AP, proteintech, USA), NDC80 (1:1000, proteintech, 18932-1-AP), BIRC5 (1:1000, #10508-1-AP, proteintech, USA), Cdc20 (1:2000, #10252-1-AP, proteintech, USA), and Tubulin (1:10000; T9026, Sigma). After primary antibody incubation, the blots were washed and incubated with the secondary anti-rabbit IgG HRP-linked antibody (1:5000; #7074, Cell Signaling Technology) at room temperature for 1 h. Then proteins were visualized by HRP substrates reactions with GE Amersham Imager (GE Healthcare, Boston, MA, USA), and the protein levels were analyzed by ImageJ software.

## Cell migration and invasion assays

To evaluate migration and invasion, we utilized separate transwell chambers. Lung cancer cells were digested and resuspended in serum-free medium. A total of 5 × 10^4^ cells were carefully seeded into the upper chamber, while the lower chamber was filled with RPMI-1640 medium supplemented with 20% Fetal Bovine Serum. Following a 24-hour incubation period, the number of migrated and invaded cells was quantified by staining and examining six distinct fields under a microscope.

## Cell counting kit-8 (CCK-8) assay

For cell viability assessment, 3 × 10^3^ cells of A549 were incubated in 96-well plates. Each well received 10 μl of CCK-8 solution mixed with 90 μl RPMI 1640 medium. Following incubation at 37°C for 2 hours, the optical density (OD) was measured at 0, 24, 48, and 72 hours to monitor cell proliferation over time.

## Cell apoptosis detection

To evaluate apoptosis in lung cancer cells, we utilized the Annexin V-FITC/PI Apoptosis Detection Kit (KeyGEN Biotech, KGA107). Upon reaching 70–80% confluence, cells were trypsinized, resuspended, and stained with 5 μl Annexin V-FITC and propidium iodide for 20 minutes in the dark. Analysis of the stained cells was conducted using FlowJo software (Becton Dickinson & Company).

# SUPPLEMENTARY REFERENCES

**1.** Charoentong P, Finotello F, Angelova M, et al. Pan-cancer Immunogenomic Analyses Reveal Genotype-Immunophenotype Relationships and Predictors of Response to Checkpoint Blockade. *Cell Rep.* Jan 3 2017;18(1):248-262.

**2.** Hanzelmann S, Castelo R, Guinney J. GSVA: gene set variation analysis for microarray and RNA-seq data. *BMC Bioinformatics.* Jan 16 2013;14:7.

**3.** Schupp JC, Adams TS, Cosme C, Jr., et al. Integrated Single-Cell Atlas of Endothelial Cells of the Human Lung. *Circulation.* Jul 27 2021;144(4):286-302.

**4.** Lambrechts D, Wauters E, Boeckx B, et al. Phenotype molding of stromal cells in the lung tumor microenvironment. *Nat Med.* Aug 2018;24(8):1277-1289.

**5.** Hu J, Bianchi F, Ferguson M, et al. Gene expression signature for angiogenic and nonangiogenic non-small-cell lung cancer. *Oncogene.* Feb 10 2005;24(7):1212-1219.

**6.** Masiero M, Simoes FC, Han HD, et al. A core human primary tumor angiogenesis signature identifies the endothelial orphan receptor ELTD1 as a key regulator of angiogenesis. *Cancer Cell.* Aug 12 2013;24(2):229-241.

**7.** Buechler MB, Pradhan RN, Krishnamurty AT, et al. Cross-tissue organization of the fibroblast lineage. *Nature.* May 2021;593(7860):575-579.

**8.** Navab R, Strumpf D, Bandarchi B, et al. Prognostic gene-expression signature of carcinoma-associated fibroblasts in non-small cell lung cancer. *Proc Natl Acad Sci U S A.* Apr 26 2011;108(17):7160-7165.

**9.** Galbo PM, Jr., Zang X, Zheng D. Molecular Features of Cancer-associated Fibroblast Subtypes and their Implication on Cancer Pathogenesis, Prognosis, and Immunotherapy Resistance. *Clin Cancer Res.* May 1 2021;27(9):2636-2647.

**10.** Moffitt RA, Marayati R, Flate EL, et al. Virtual microdissection identifies distinct tumor- and stroma-specific subtypes of pancreatic ductal adenocarcinoma. *Nat Genet.* Oct 2015;47(10):1168-1178.

**11.** Chakravarthy A, Khan L, Bensler NP, Bose P, De Carvalho DD. TGF-beta-associated extracellular matrix genes link cancer-associated fibroblasts to immune evasion and immunotherapy failure. *Nat Commun.* Nov 8 2018;9(1):4692.

**12.** Shao X, Taha IN, Clauser KR, Gao YT, Naba A. MatrisomeDB: the ECM-protein knowledge database. *Nucleic Acids Res.* Jan 8 2020;48(D1):D1136-D1144.

**13.** Yoshihara K, Shahmoradgoli M, Martinez E, et al. Inferring tumour purity and stromal and immune cell admixture from expression data. *Nat Commun.* 2013;4:2612.

**14.** Bagaev A, Kotlov N, Nomie K, et al. Conserved pan-cancer microenvironment subtypes predict response to immunotherapy. *Cancer Cell.* Jun 14 2021;39(6):845-865 e847.

**15.** Bindea G, Mlecnik B, Tosolini M, et al. Spatiotemporal dynamics of intratumoral immune cells reveal the immune landscape in human cancer. *Immunity.* Oct 17 2013;39(4):782-795.

**16.** Davoli T, Uno H, Wooten EC, Elledge SJ. Tumor aneuploidy correlates with markers of immune evasion and with reduced response to immunotherapy. *Science.* Jan 20 2017;355(6322).

**17.** Heng TS, Painter MW, Immunological Genome Project C. The Immunological Genome Project: networks of gene expression in immune cells. *Nat Immunol.* Oct 2008;9(10):1091-1094.

**18.** Zeng D, Ye Z, Shen R, et al. IOBR: Multi-Omics Immuno-Oncology Biological Research to Decode Tumor Microenvironment and Signatures. *Front Immunol.* 2021;12:687975.

**19.** Newman AM, Steen CB, Liu CL, et al. Determining cell type abundance and expression from bulk tissues with digital cytometry. *Nat Biotechnol.* Jul 2019;37(7):773-782.

**20.** Racle J, de Jonge K, Baumgaertner P, Speiser DE, Gfeller D. Simultaneous enumeration of cancer and immune cell types from bulk tumor gene expression data. *Elife.* Nov 13 2017;6.

**21.** Becht E, Giraldo NA, Lacroix L, et al. Estimating the population abundance of tissue-infiltrating immune and stromal cell populations using gene expression. *Genome Biol.* Oct 20 2016;17(1):218.

**22.** Finotello F, Mayer C, Plattner C, et al. Molecular and pharmacological modulators of the tumor immune contexture revealed by deconvolution of RNA-seq data. *Genome Med.* May 24 2019;11(1):34.

**23.** Li B, Severson E, Pignon JC, et al. Comprehensive analyses of tumor immunity: implications for cancer immunotherapy. *Genome Biol.* Aug 22 2016;17(1):174.

**24.** Aran D, Hu Z, Butte AJ. xCell: digitally portraying the tissue cellular heterogeneity landscape. *Genome Biol.* Nov 15 2017;18(1):220.

**25.** Mariathasan S, Turley SJ, Nickles D, et al. TGFbeta attenuates tumour response to PD-L1 blockade by contributing to exclusion of T cells. *Nature.* Feb 22 2018;554(7693):544-548.

**26.** Tang H, Xiao G, Behrens C, et al. A 12-gene set predicts survival benefits from adjuvant chemotherapy in non-small cell lung cancer patients. *Clin Cancer Res.* Mar 15 2013;19(6):1577-1586.

**27.** Guo NL, Dowlati A, Raese RA, et al. A Predictive 7-Gene Assay and Prognostic Protein Biomarkers for Non-small Cell Lung Cancer. *EBioMedicine.* Jun 2018;32:102-110.

**28.** Lim SB, Tan SJ, Lim WT, Lim CT. An extracellular matrix-related prognostic and predictive indicator for early-stage non-small cell lung cancer. *Nat Commun.* Nov 23 2017;8(1):1734.

**29.** Angulo B, Suarez-Gauthier A, Lopez-Rios F, et al. Expression signatures in lung cancer reveal a profile for EGFR-mutant tumours and identify selective PIK3CA overexpression by gene amplification. *J Pathol.* Feb 2008;214(3):347-356.

**30.** Shibata T, Hanada S, Kokubu A, et al. Gene expression profiling of epidermal growth factor receptor/KRAS pathway activation in lung adenocarcinoma. *Cancer Sci.* Jul 2007;98(7):985-991.

**31.** Balko JM, Potti A, Saunders C, Stromberg A, Haura EB, Black EP. Gene expression patterns that predict sensitivity to epidermal growth factor receptor tyrosine kinase inhibitors in lung cancer cell lines and human lung tumors. *BMC Genomics.* Nov 10 2006;7:289.

**32.** Coldren CD, Helfrich BA, Witta SE, et al. Baseline gene expression predicts sensitivity to gefitinib in non-small cell lung cancer cell lines. *Mol Cancer Res.* Aug 2006;4(8):521-528.

**33.** Okayama H, Kohno T, Ishii Y, et al. Identification of genes upregulated in ALK-positive and EGFR/KRAS/ALK-negative lung adenocarcinomas. *Cancer Res.* Jan 1 2012;72(1):100-111.

**34.** Franzini A, Baty F, Macovei, II, et al. Gene Expression Signatures Predictive of Bevacizumab/Erlotinib Therapeutic Benefit in Advanced Nonsquamous Non-Small Cell Lung Cancer Patients (SAKK 19/05 trial). *Clin Cancer Res.* Dec 1 2015;21(23):5253-5263.

**35.** Haitang Yang BS, Sean R R Hall, Ke Xu, Liang Zhao, Swee T. Tan, Ralph A. Schmid, Ren-Wang Peng, Feng Yao. Responsive signatures established by pharmaco-transcriptomic correlation analysis identifies subsets for PARP-targeted therapy and reveals potential synergistic interactors. *The 1st International Electronic Conference on Cancers: Exploiting Cancer Vulnerability by Targeting the DNA Damage Response.* 2021:1-14.

**36.** Ayers M, Lunceford J, Nebozhyn M, et al. IFN-gamma-related mRNA profile predicts clinical response to PD-1 blockade. *J Clin Invest.* Aug 1 2017;127(8):2930-2940.

**37.** Wang H, Li S, Wang Q, et al. Tumor immunological phenotype signature-based high-throughput screening for the discovery of combination immunotherapy compounds. *Sci Adv.* Jan 2021;7(4).

**38.** Thompson JC, Davis C, Deshpande C, et al. Gene signature of antigen processing and presentation machinery predicts response to checkpoint blockade in non-small cell lung cancer (NSCLC) and melanoma. *J Immunother Cancer.* Oct 2020;8(2).

**39.** Hugo W, Zaretsky JM, Sun L, et al. Genomic and Transcriptomic Features of Response to Anti-PD-1 Therapy in Metastatic Melanoma. *Cell.* Mar 24 2016;165(1):35-44.

**40.** Calon A, Espinet E, Palomo-Ponce S, et al. Dependency of colorectal cancer on a TGF-beta-driven program in stromal cells for metastasis initiation. *Cancer Cell.* Nov 13 2012;22(5):571-584.

**41.** Lu Z, Li Y, Che Y, et al. The TGFbeta-induced lncRNA TBILA promotes non-small cell lung cancer progression in vitro and in vivo via cis-regulating HGAL and activating S100A7/JAB1 signaling. *Cancer Lett.* Sep 28 2018;432:156-168.

**42.** Lachenmayer A, Alsinet C, Savic R, et al. Wnt-pathway activation in two molecular classes of hepatocellular carcinoma and experimental modulation by sorafenib. *Clin Cancer Res.* Sep 15 2012;18(18):4997-5007.

**43.** Bild AH, Yao G, Chang JT, et al. Oncogenic pathway signatures in human cancers as a guide to targeted therapies. *Nature.* Jan 19 2006;439(7074):353-357.

**44.** Rees MG, Seashore-Ludlow B, Cheah JH, et al. Correlating chemical sensitivity and basal gene expression reveals mechanism of action. *Nat Chem Biol.* Feb 2016;12(2):109-116.

**45.** Corsello SM, Nagari RT, Spangler RD, et al. Discovering the anti-cancer potential of non-oncology drugs by systematic viability profiling. *Nat Cancer.* Feb 2020;1(2):235-248.

**46.** Yang W, Soares J, Greninger P, et al. Genomics of Drug Sensitivity in Cancer (GDSC): a resource for therapeutic biomarker discovery in cancer cells. *Nucleic Acids Res.* Jan 2013;41(Database issue):D955-961.

**47.** Maeser D, Gruener RF, Huang RS. oncoPredict: an R package for predicting in vivo or cancer patient drug response and biomarkers from cell line screening data. *Brief Bioinform.* Nov 5 2021;22(6).

# SUPPLEMENTARY FIGURES

**
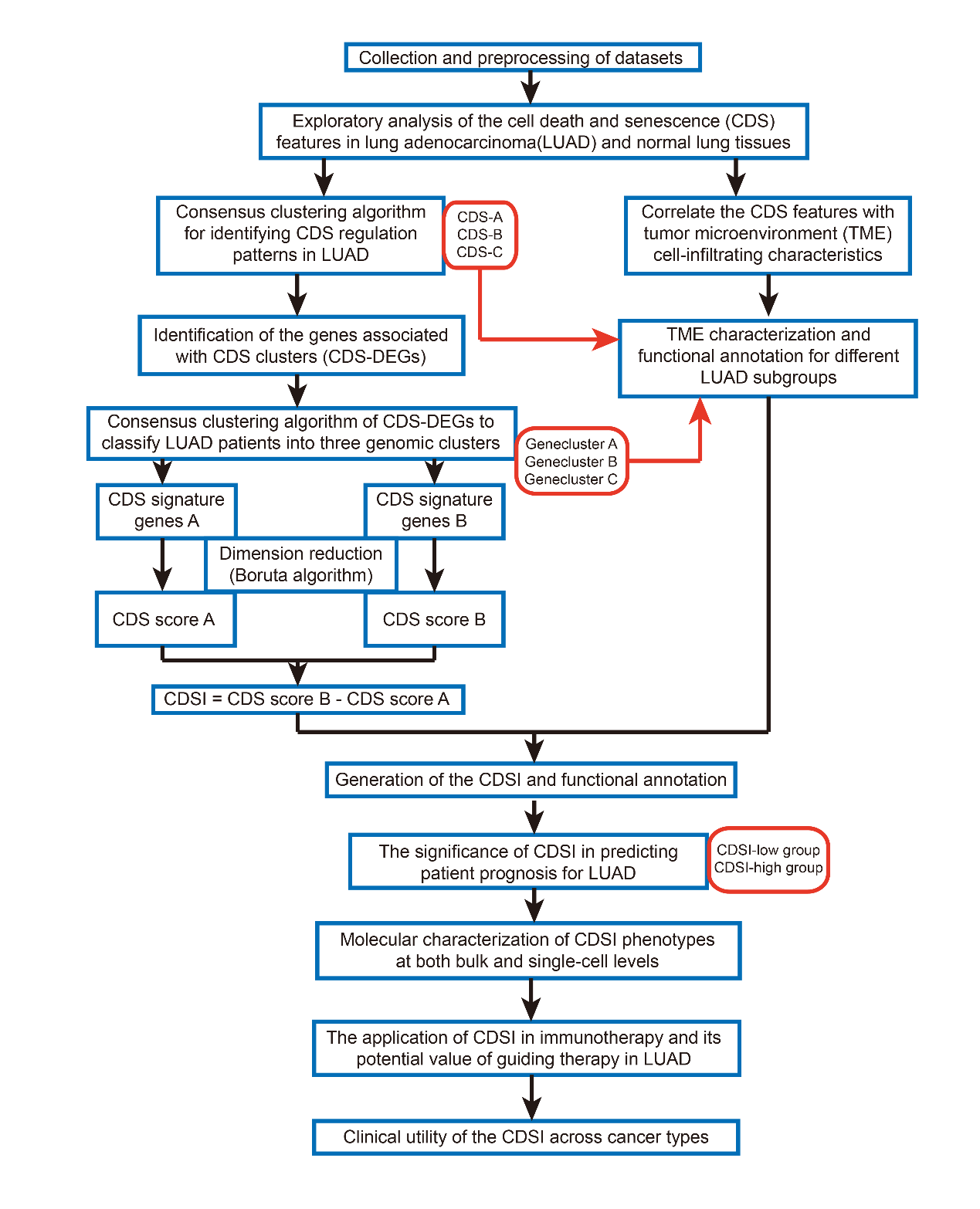
**

**Figure S1.** Overview of the study design.


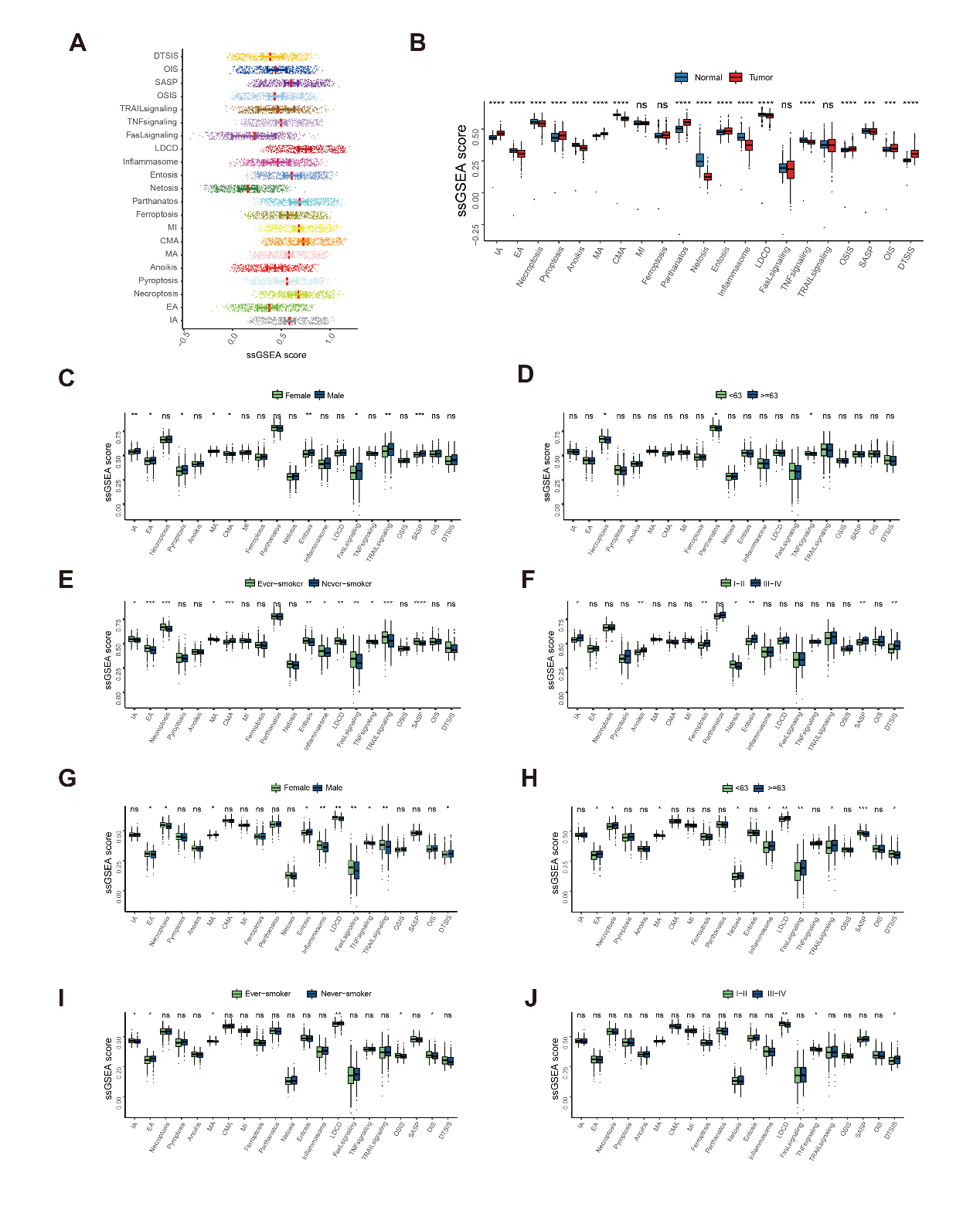


**Figure S2.** Activities of CDS features in LUAD. **A** Distribution of the CDS activities in the LUAD samples from TCGA cohort. **B** Difference in the CDS activities between LUAD and normal lung samples using TCGA data. **C–F** Differences in the CDS activities between subgroups of LUAD stratified on the basis of sex (**C**), age (**D**), smoking (**E**), and stages (**F**) in the LuMMD cohort. **G–J** Differences in the CDS activities between subgroups of LUAD stratified on the basis of sex (**G**), age (**H**), smoking (**I**), and stages (**J**) in TCGA cohort. In **B–J**, the upper and lower ends of the boxes represent the interquartile range of values, the lines in the boxes represent median values, and black dots show outliers. P values were calculated using the Wilcoxon rank-sum test. Ns, not significant; *p < 0.05; **p ≤ 0.01; ***p ≤ 0.001; ****p ≤ 0.0001.


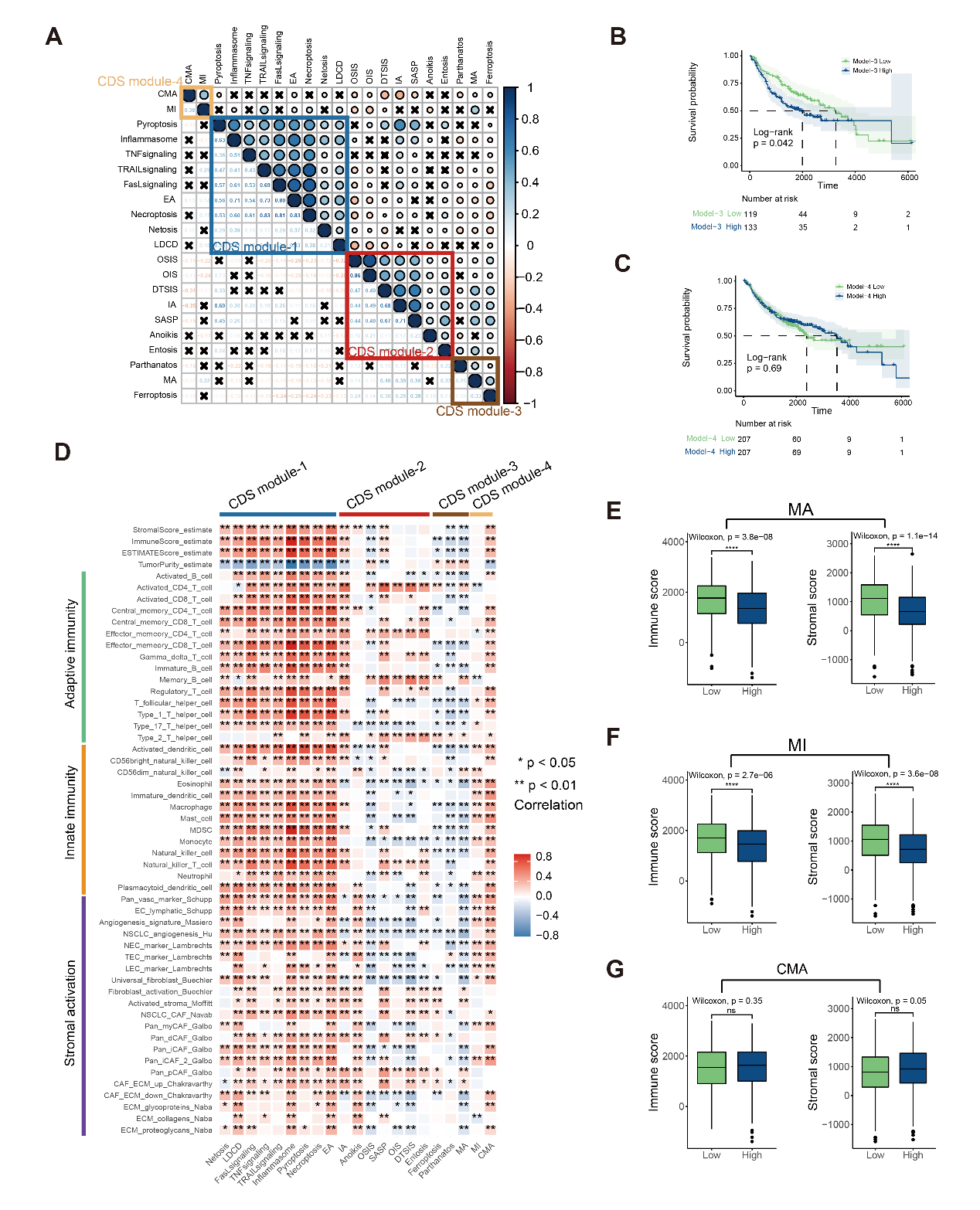


**Figure S3.** Association between CDS activities and TME characteristics in LUAD. **A** Four CDS modules were identified based on the Spearman correlations between the CDS features. Detailed results are provided in Table S6. **B-C** Kaplan–Meier curves of OS for LuMMD patients stratified by activities of module-3 (**B**) and -4 (**C**). The p-values were calculated using the log-rank test. **D** Correlation between each CDS feature and each TME-infiltrating cell type using Spearman analyses and TCGA data. Positive and negative correlations are marked with red and blue, respectively. **E–G** Difference in the immune and stromal scores in LUAD between low and high CDS activities groups. Analyses were performed for MA (**E**), MI (**F**), and CMA (**G**). In **E–G**, the upper and lower ends of the boxes represent the interquartile range of values, the lines in the boxes represent median values, and black dots show outliers. P values were tested using the Wilcoxon rank-sum test. Ns, not significant; *p < 0.05; **p ≤ 0.01; ****p ≤ 0.0001.


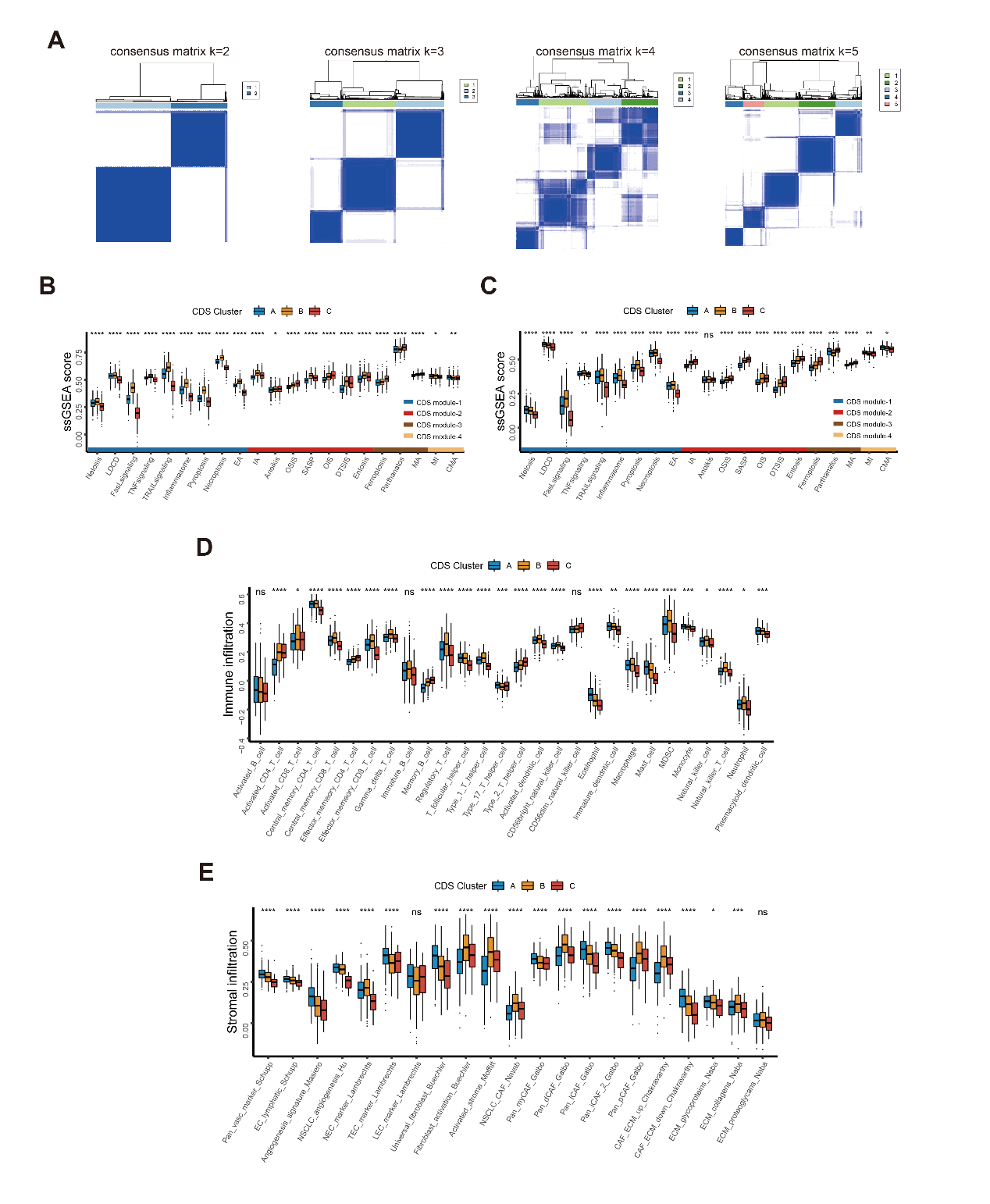


**Figure S4.** Consensus clustering for CDS subtypes and related CDS activities, TME characteristics of each CDS phenotype. **A** Consensus matrixes of the LuMMD cohort for each k (k = 2–5), displaying the clustering stability of the CDS activities using 1000 iterations of hierarchical clustering. **B** Activities of 21 CDS features in three CDS subtypes based on the LuMMD cohort. The CDS features belong to four modules. **C** Activities of 21 CDS features in three CDS subtypes based on TCGA cohort. **D, E** TME-infiltrating (**D**) immune and (**E**) stromal cells in three CDS subtypes based on TCGA cohort. The upper and lower ends of the boxes in **B**-**E** represent the interquartile range of values, the lines in the boxes represent median values, and black dots show outliers. P values were tested using the Kruskal–Wallis test. Ns, not significant; *p < 0.05; **p ≤ 0.01; ***p ≤ 0.001; ****p ≤ 0.0001.


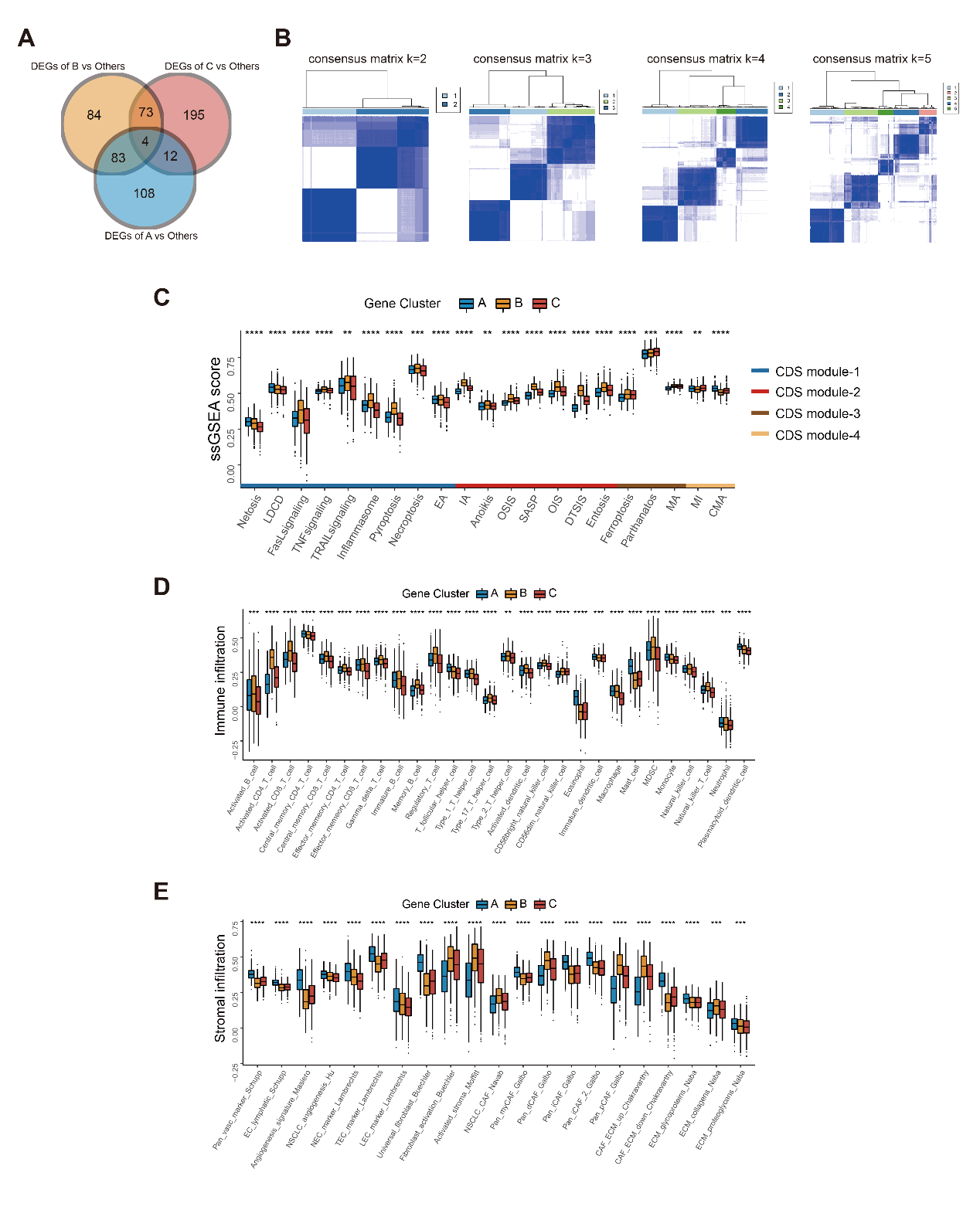


**Figure S5.** Consensus clustering for CDS genomic subtypes and related CDS activities, TME characteristics in each genomic subtype. **A** Venn diagram illustrating the number of DEGs among the three CDS subtypes. **B** Consensus matrixes of the LuMMD cohort for each k (k = 2–5), displaying the clustering stability of the CDS-associated DEGs using 1000 iterations of hierarchical clustering. **C** Activities of 21 CDS features in three genomic clusters based on the LuMMD cohort. These CDS features belong to four modules. **D, E** TME-infiltrating (**D**) immune and (**E**) stromal cells in three genomic clusters based on the LuMMD cohort. In **C–E**, the upper and lower ends of the boxes represent the interquartile range of values, the lines in the boxes represent median values, and black dots show outliers. P values were tested using the Kruskal–Wallis test. Ns, not significant; *p < 0.05; **p ≤ 0.01; ***p ≤ 0.001; ****p ≤ 0.0001.


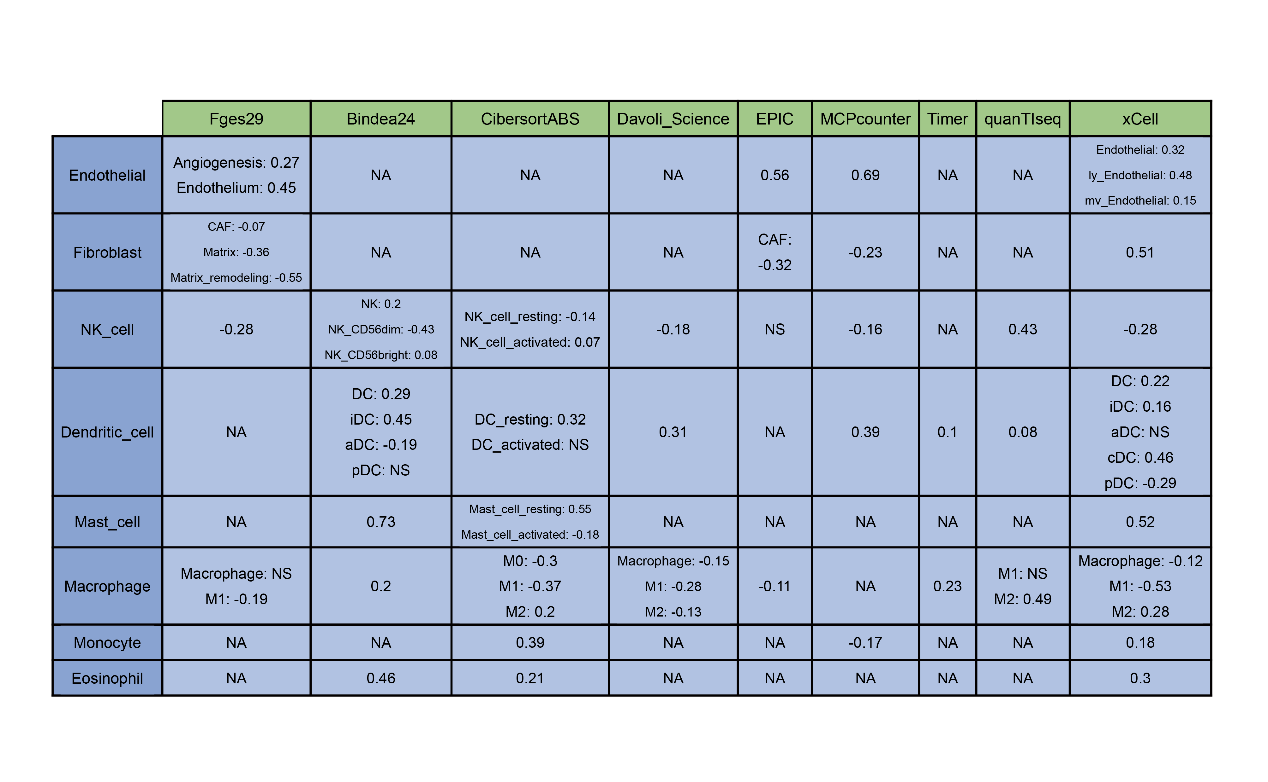


**Figure S6.** Association of CDSI with TME-infiltrating cells in the LuMMD cohort. Spearman correlation between CDSI and infiltration levels of eight types of TME-infiltrating cells that were calculated using nine independent algorithms and gene signatures.


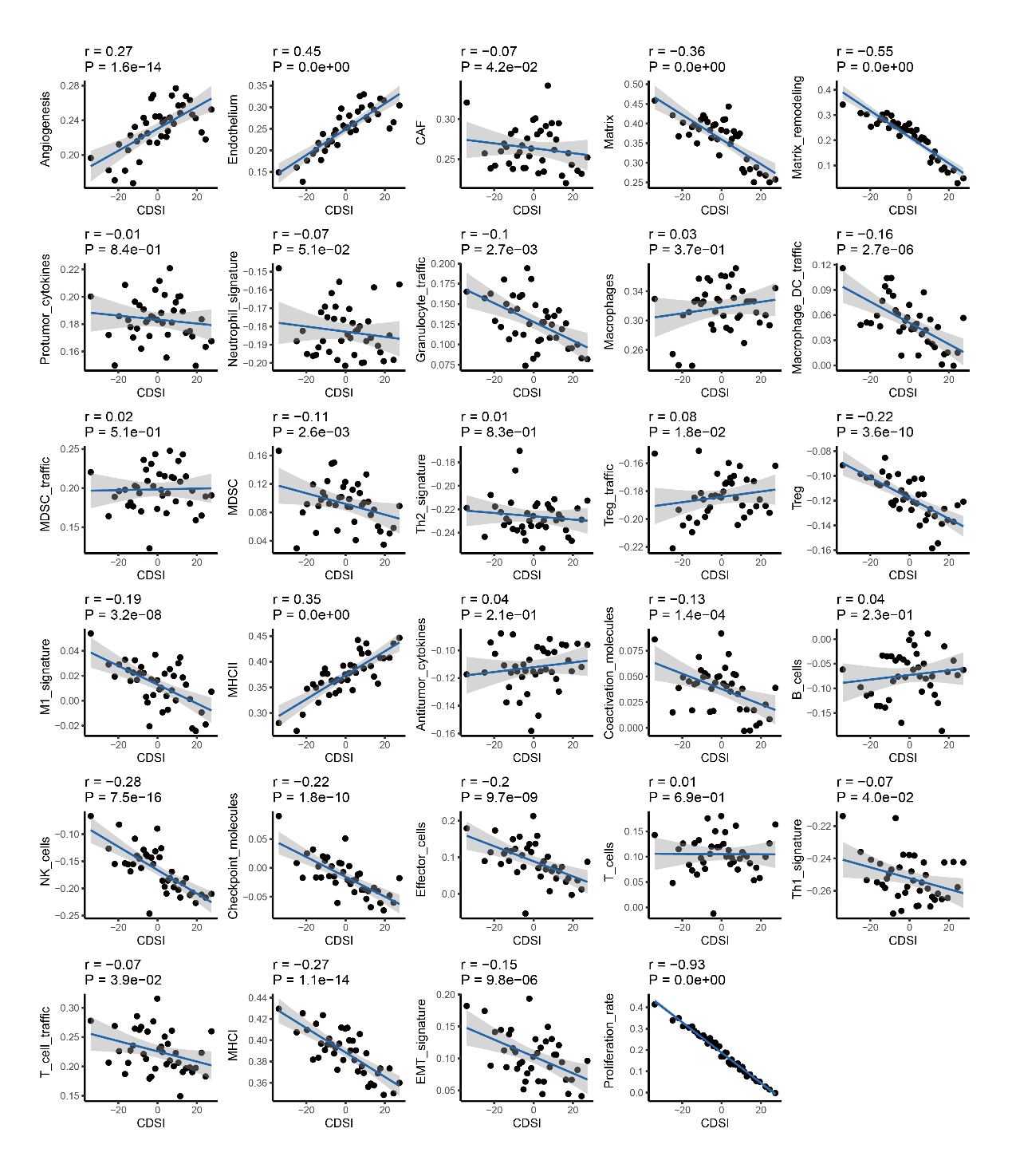


**Figure S7.** Correlation between CDSI and each of the 29 TME-infiltrating cell types curated by Bagaev A using Spearman analyses. The infiltration levels of these cell types were calculated using ssGSEA.


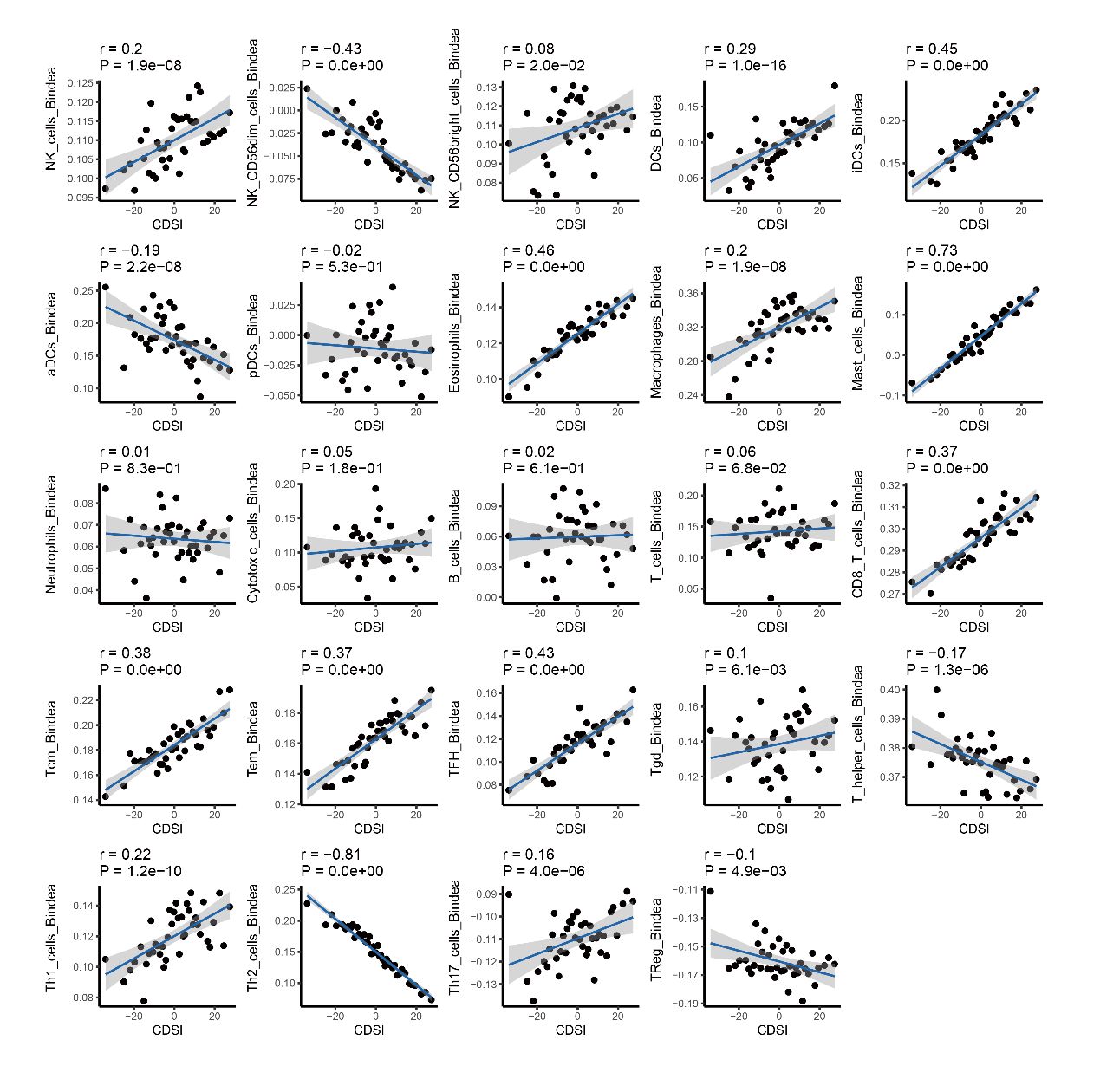


**Figure S8.** Correlation between CDSI and each of the 28 tumor-infiltrating immune cell types curated by Bindea G using Spearman analyses. The infiltration levels of these cell types were calculated using ssGSEA.


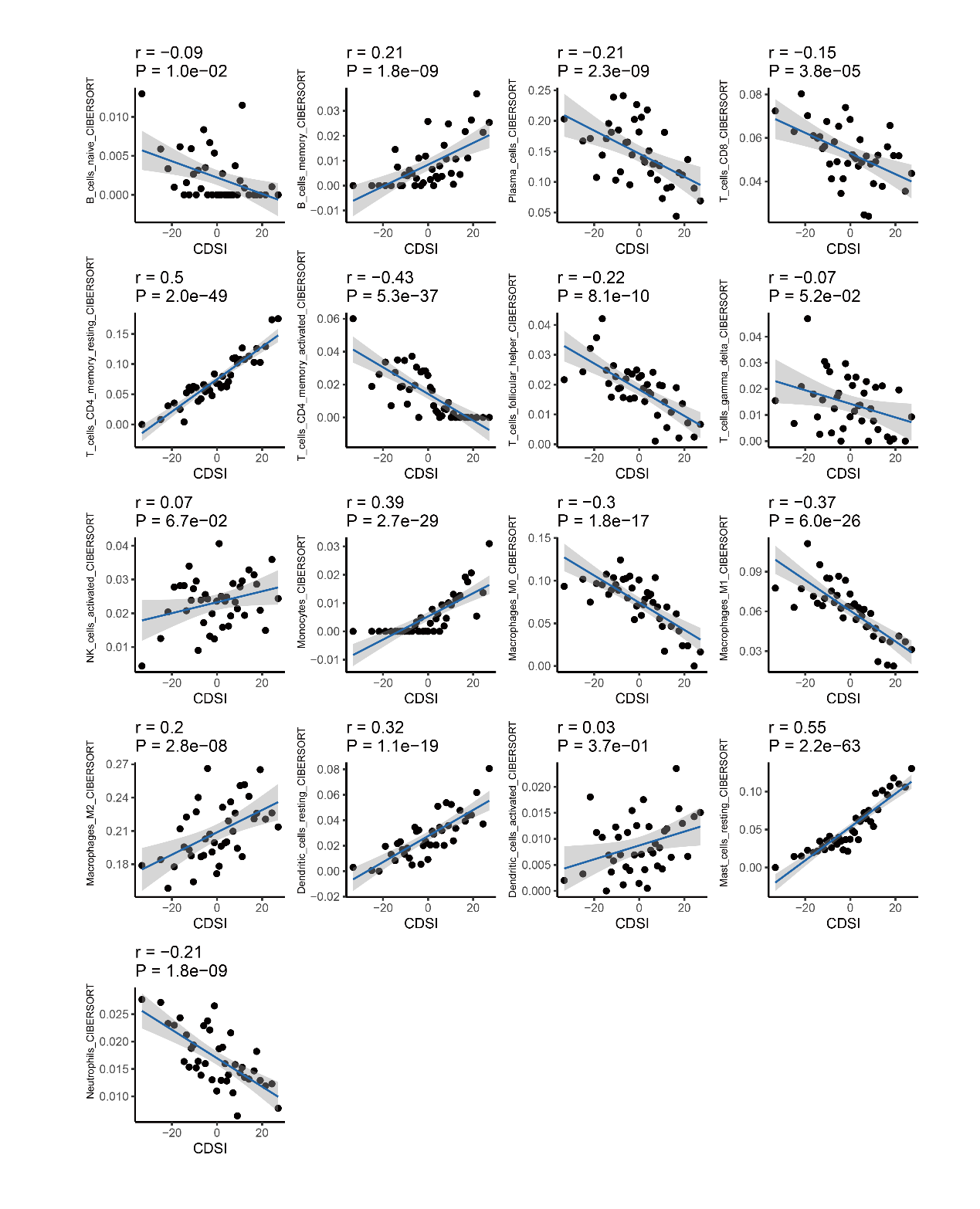


**Figure S9.** Correlation between CDSI and the infiltration levels of tumor immune cells quantified using CIBERSORT-ABS. The p value was calculated using the Spearman correlation analysis.


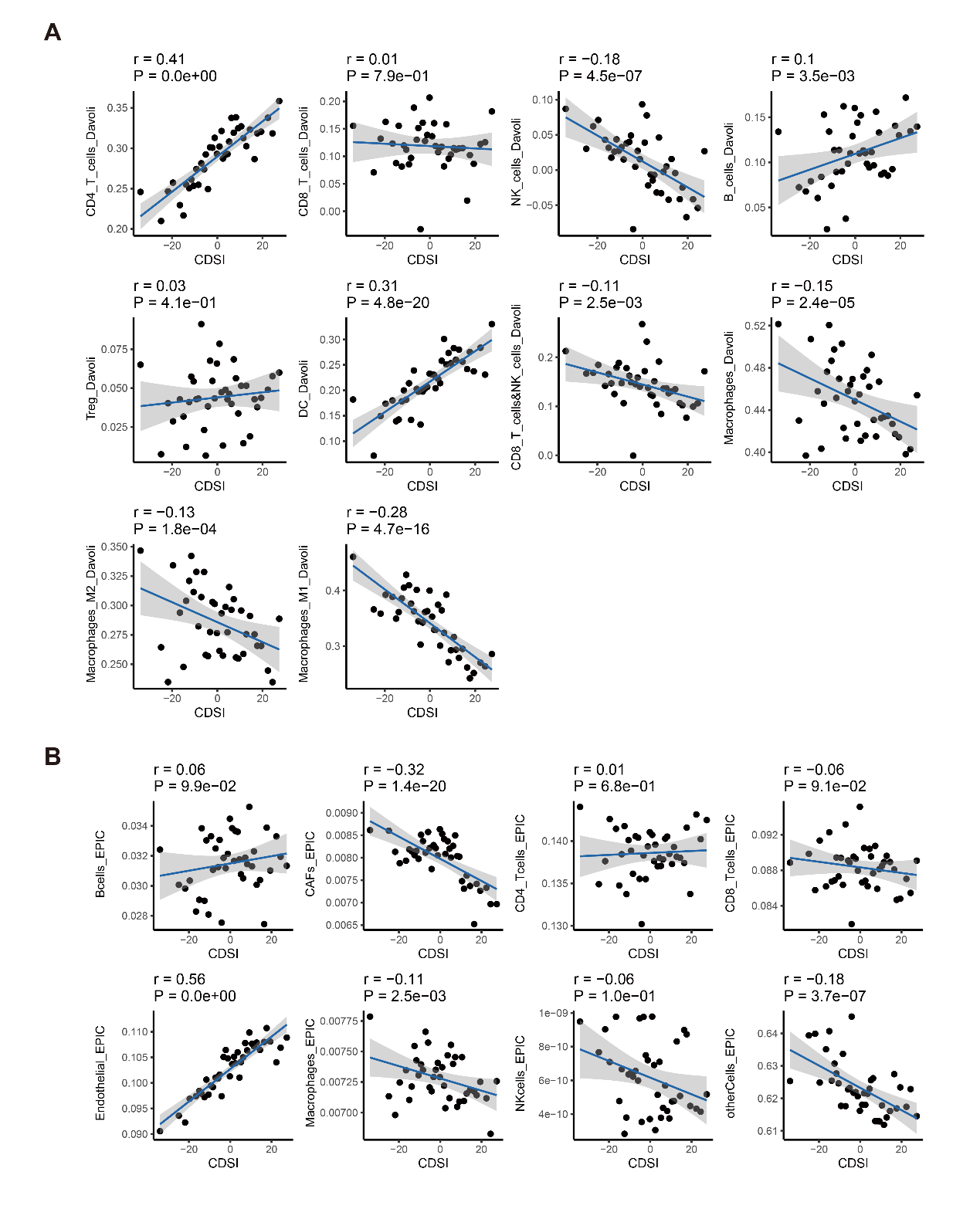


**Figure S10.** Correlation between CDSI and the infiltration levels of tumor immune cells quantified using ssGSEA and EPIC. **A** The tumor immune cell types were curated by Davoli T, and their infiltration levels were calculated using ssGSEA. **B** The infiltration levels of the immune cells were calculated using EPIC. The p value was calculated using the Spearman correlation analysis.


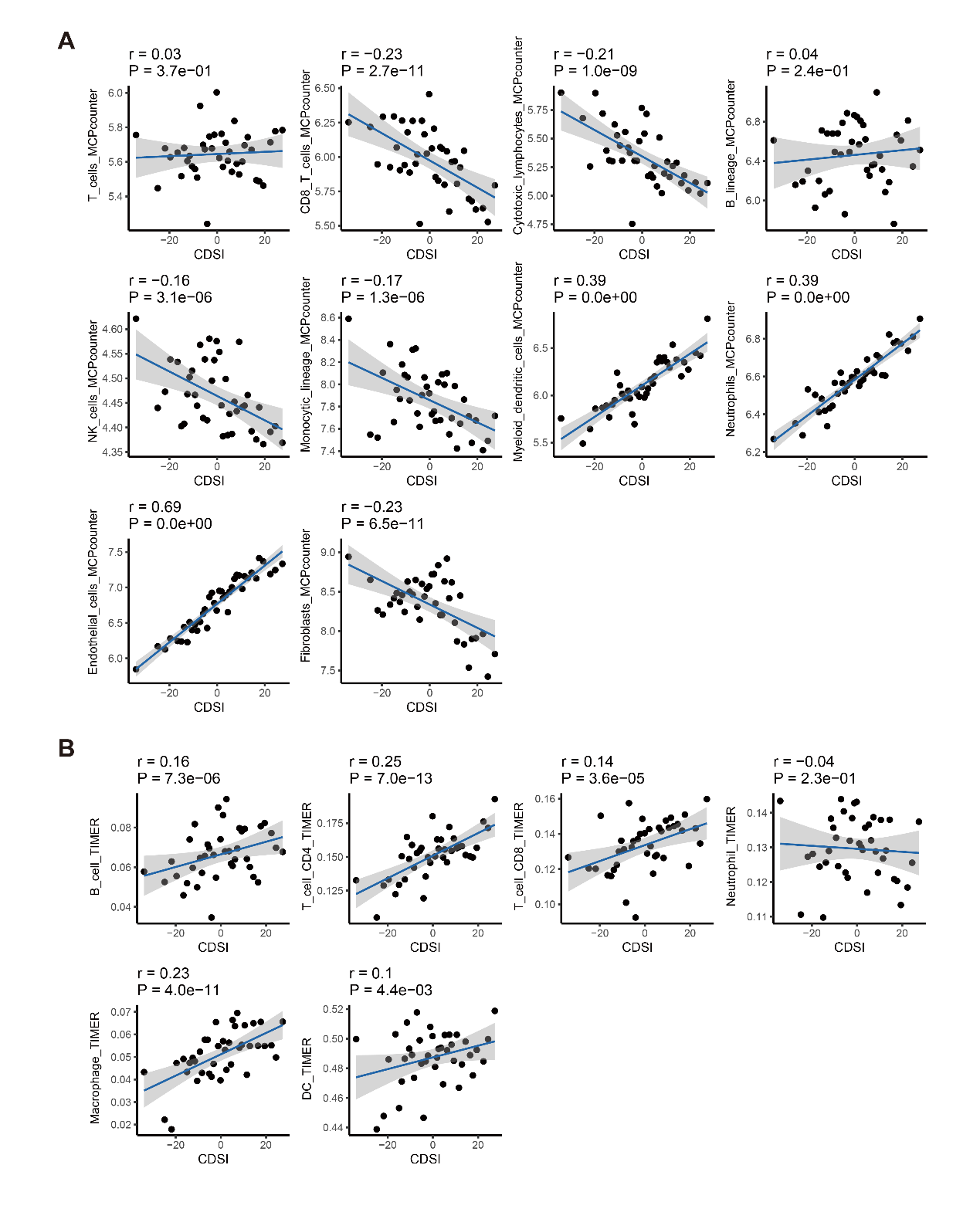


**Figure S11.** Correlation between CDSI and the infiltration levels of tumor immune cells quantified using MCP-Counter and TIMER. **A** The infiltration levels of the immune cells were calculated using MCP-Counter. **B** The infiltration levels of the immune cells were calculated using TIMER. The p value was calculated using the Spearman correlation analysis.


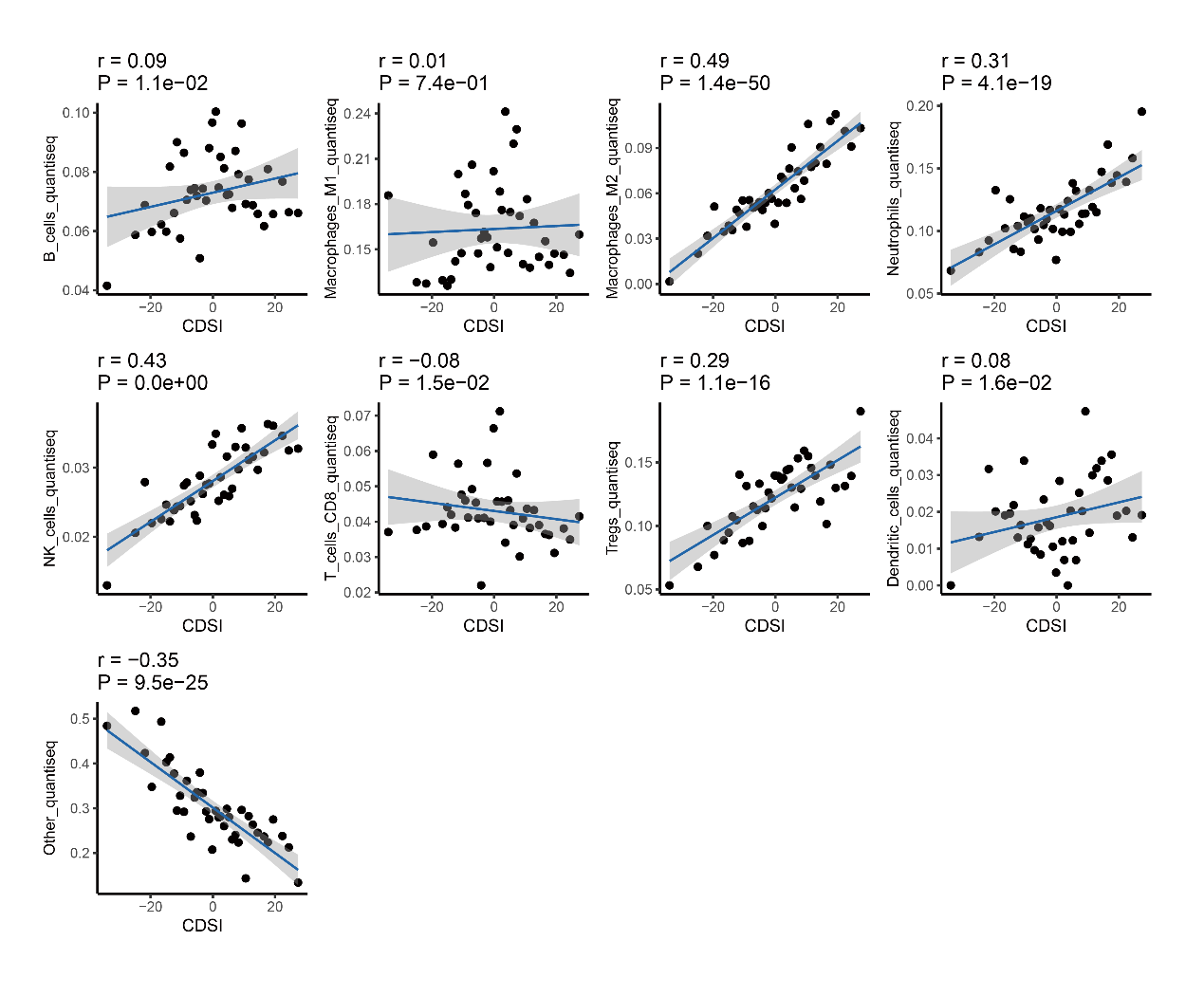


**Figure S12.** Correlation between CDSI and the infiltration levels of tumor immune cells quantified using quanTIseq. The p value was calculated using the Spearman correlation analysis.


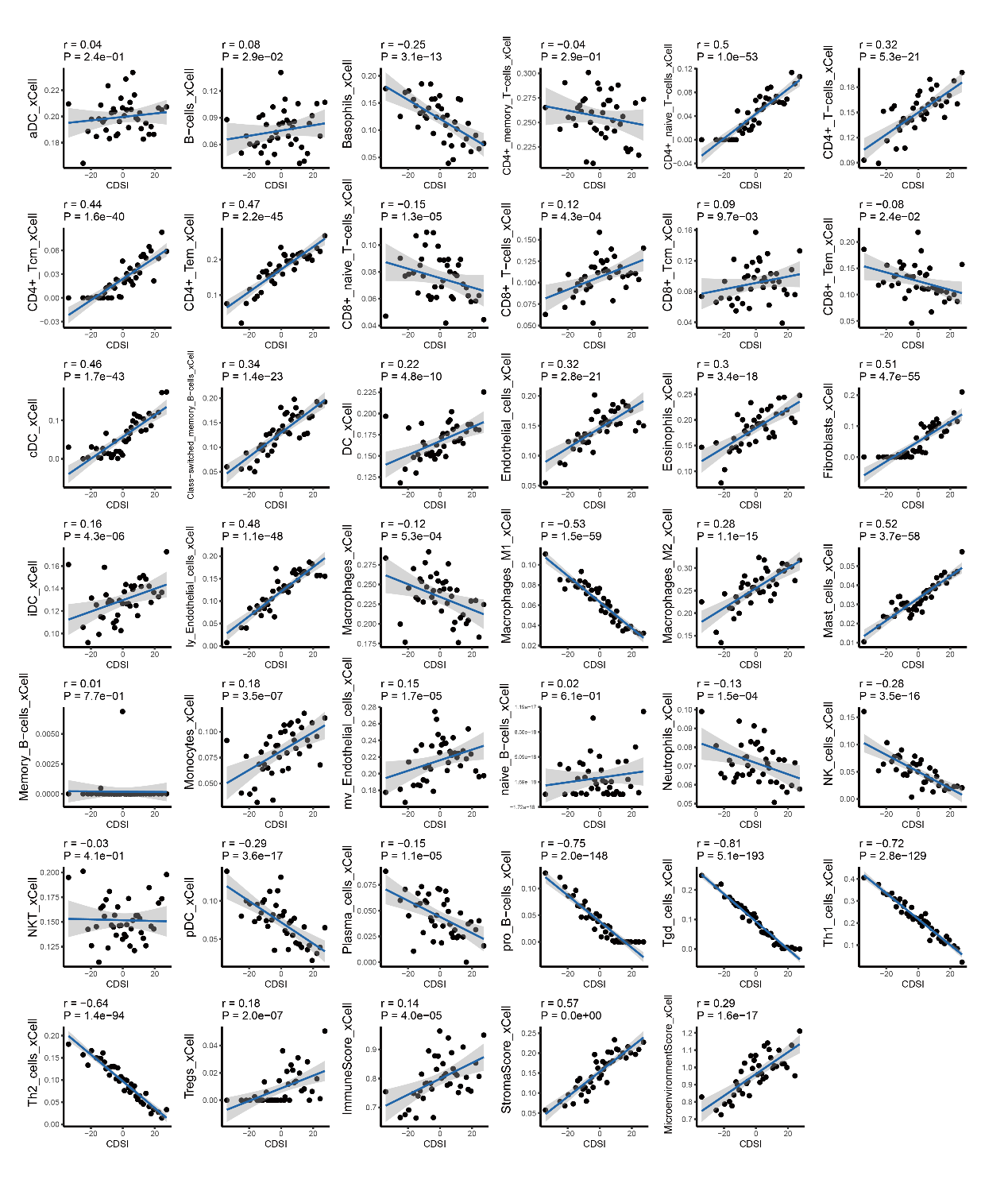


**Figure S13.** Correlation between CDSI and the infiltration levels of tumor immune cells quantified using xCell. The p value was calculated using the Spearman correlation analysis.


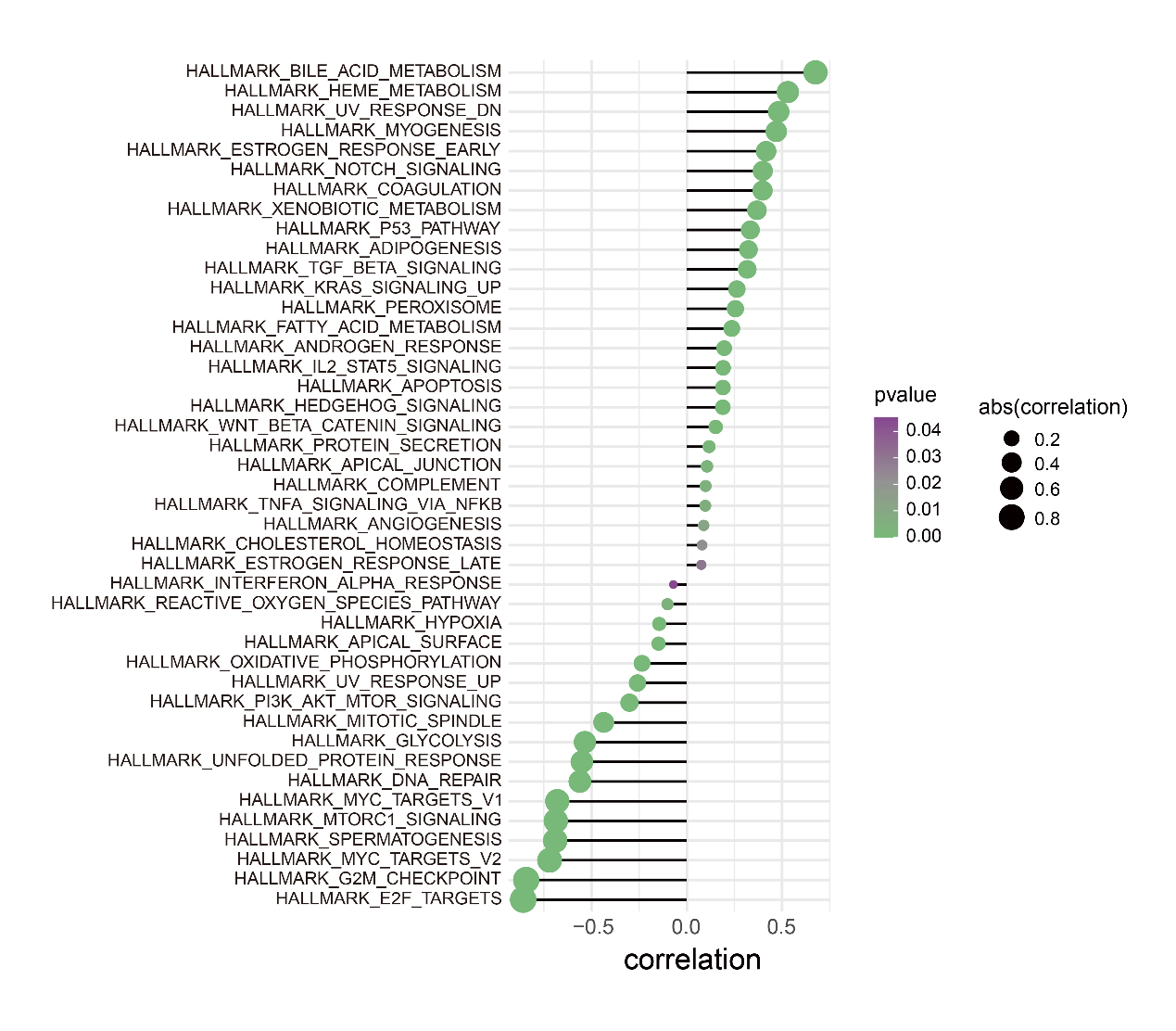


**Figure S14.** Correlations between CDSI and cancer hallmark pathways based on the LuMMD.


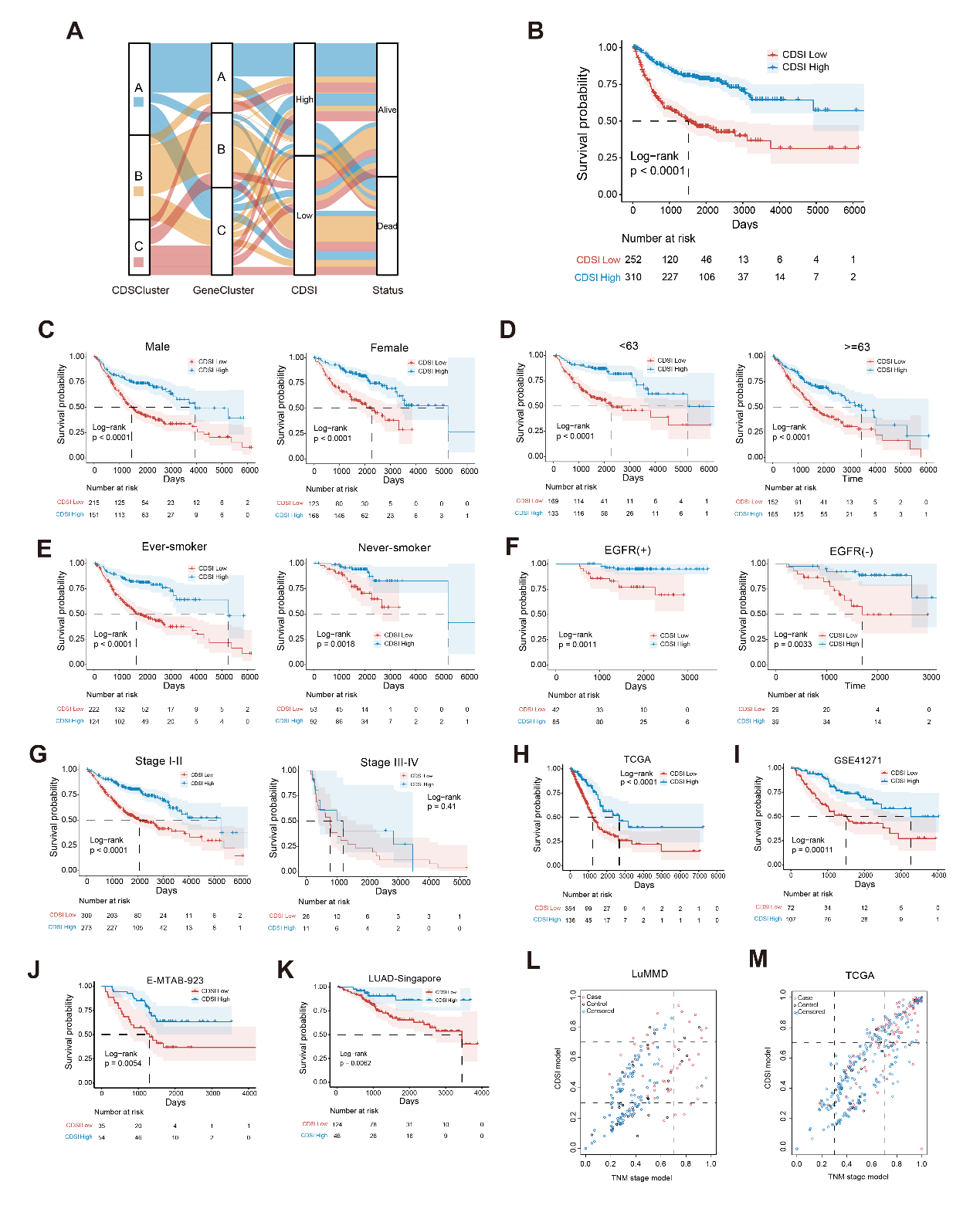


**Figure S15.** Stratified and independent validation of prognostic value of CDSI in patients with LUAD. **A** Alluvial diagram of CDS cluster distribution in groups with different gene clusters, CDSI, and survival outcomes. **B** Kaplan–Meier analysis of DFS associated with high and low CDSI subtype in the LuMMD cohort. **C–G** Kaplan–Meier curves of OS for CDSI high and low groups in patients with LUAD, stratified based on sex, age, smoking status, *EGFR* status, and TNM stage. **H–K** Kaplan–Meier curves of OS for CDSI high and low groups in patients with LUAD from independent validation cohorts, including TCGA (**H**), GSE41271 (**I**), E-MTAB-923 (**J**), and LUAD-Asia (**K**). All p values were tested using a log-rank test. **L-M** Results of net reclassification index analysis based on LuMMD (**L**) and TCGA (**M**) cohorts.


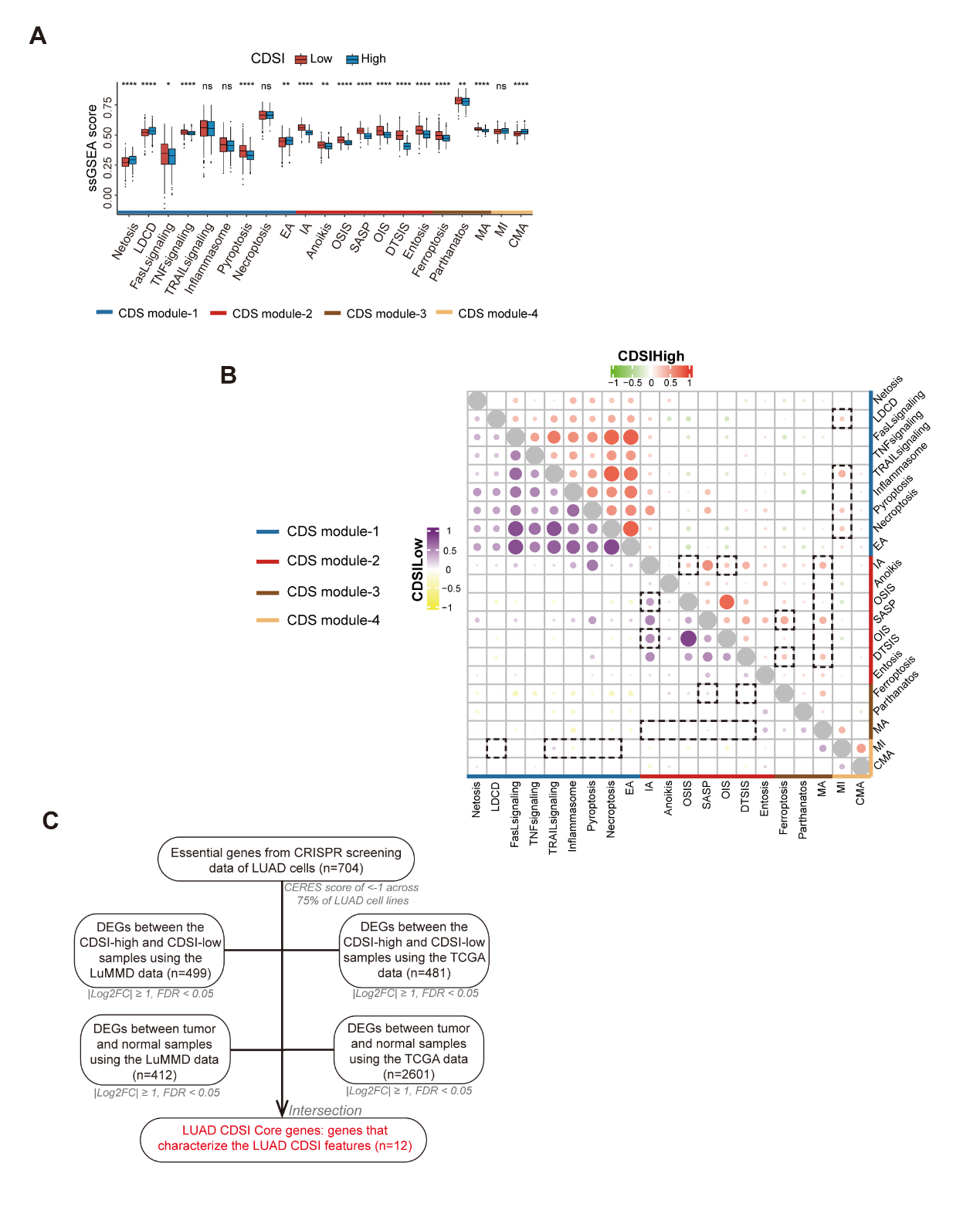


**Figure S16.** Characterization of the CDSI phenotypes. **A** Differences in CDS activities between the CDSI-high and -low groups. The upper and lower ends of the boxes represent interquartile range of values. The lines in the boxes represent median values, and black dots show outliers. P-values were calculated using the Wilcoxon rank-sum test. Ns, not significant; **p ≤ 0.01; ***p ≤ 0.001; ****p ≤ 0.0001. **B** Correlations between CDS features in the CDSI-high and -low patients with LUAD via Spearman analysis. **C** Schematic outlining the strategy to identify core genes that characterize the CDSI phenotypes.


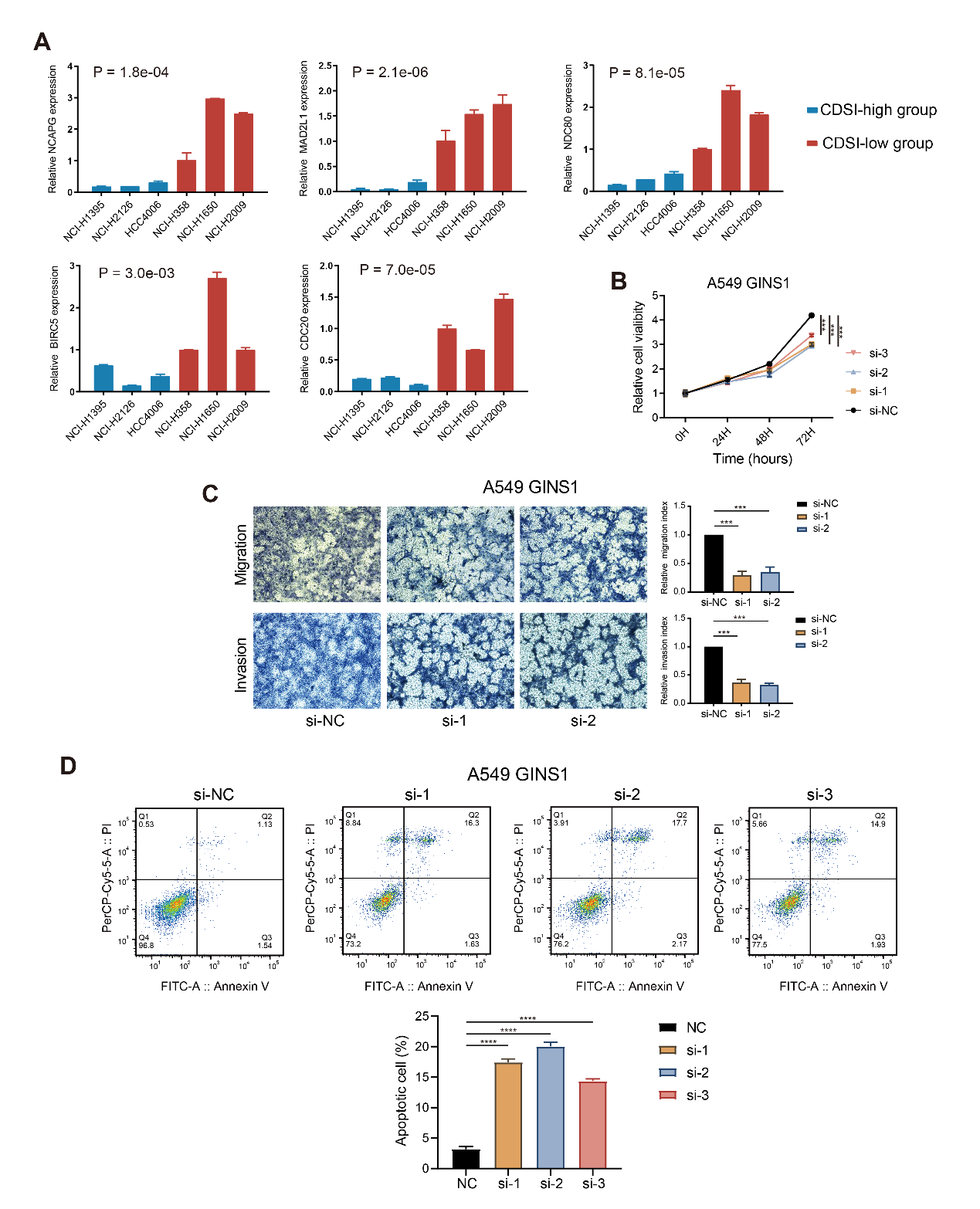


**Figure S17.** *In vitro* study of the CDSI core genes. **A** Comparison of expression of selected CDSI core genes in different CDSI cell subsets by RT-qPCR. Data represented with mean ± SD. **B** CCK-8 assay performed in A549 cell line transfected with control siRNA or *GINS1* siRNA. **C** Transwell assay performed in A549 cell line transfected with control siRNA or *GINS1* siRNA. **D** Apoptosis was determined using fluorescence activated cell sorting (FACS) analysis by Annexin V-FITC and propidium iodide (PI) co-staining, and Annexin V+ cell populations were defined as apoptotic. ***p ≤ 0.001; ****p ≤ 0.0001.

**
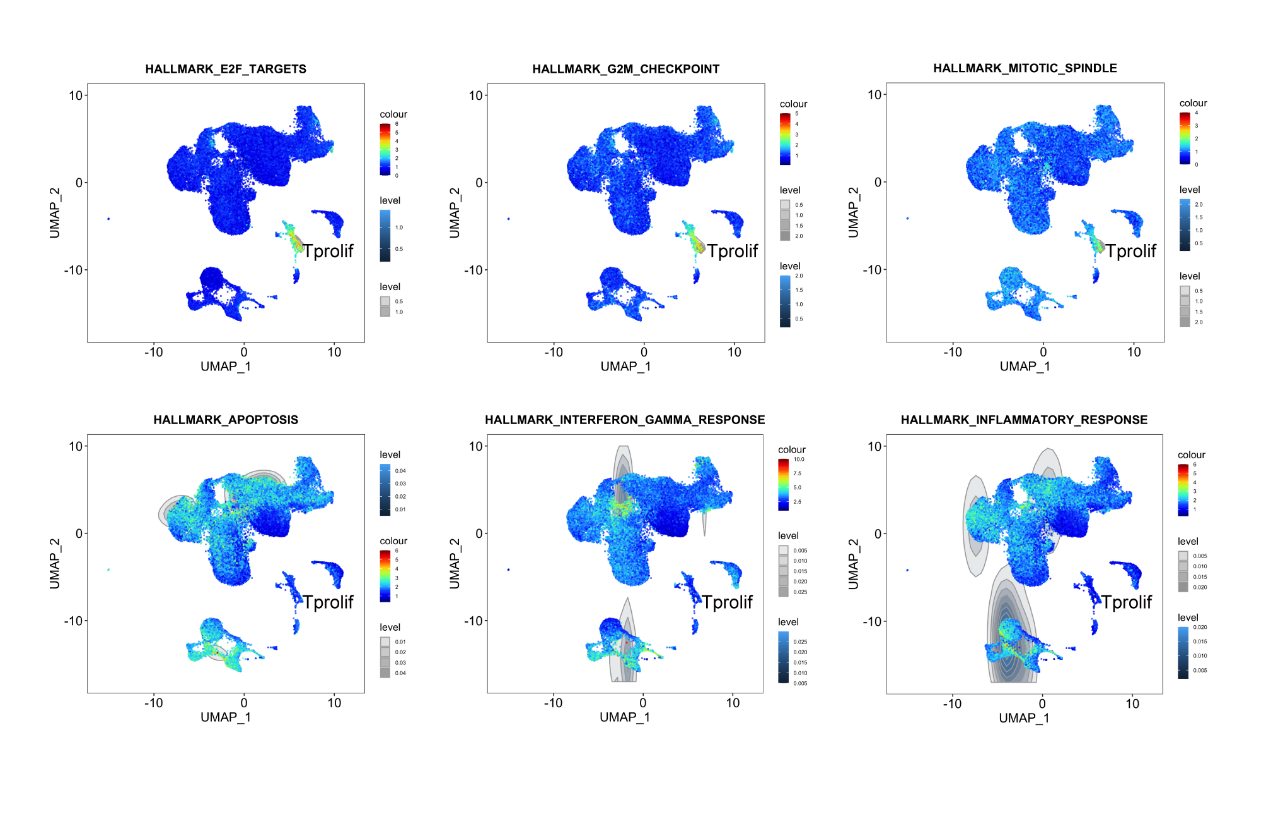
**

**Figure S18.** Single-cell GSEA analysis using cancer hallmarks based on the GSE139555 dataset.


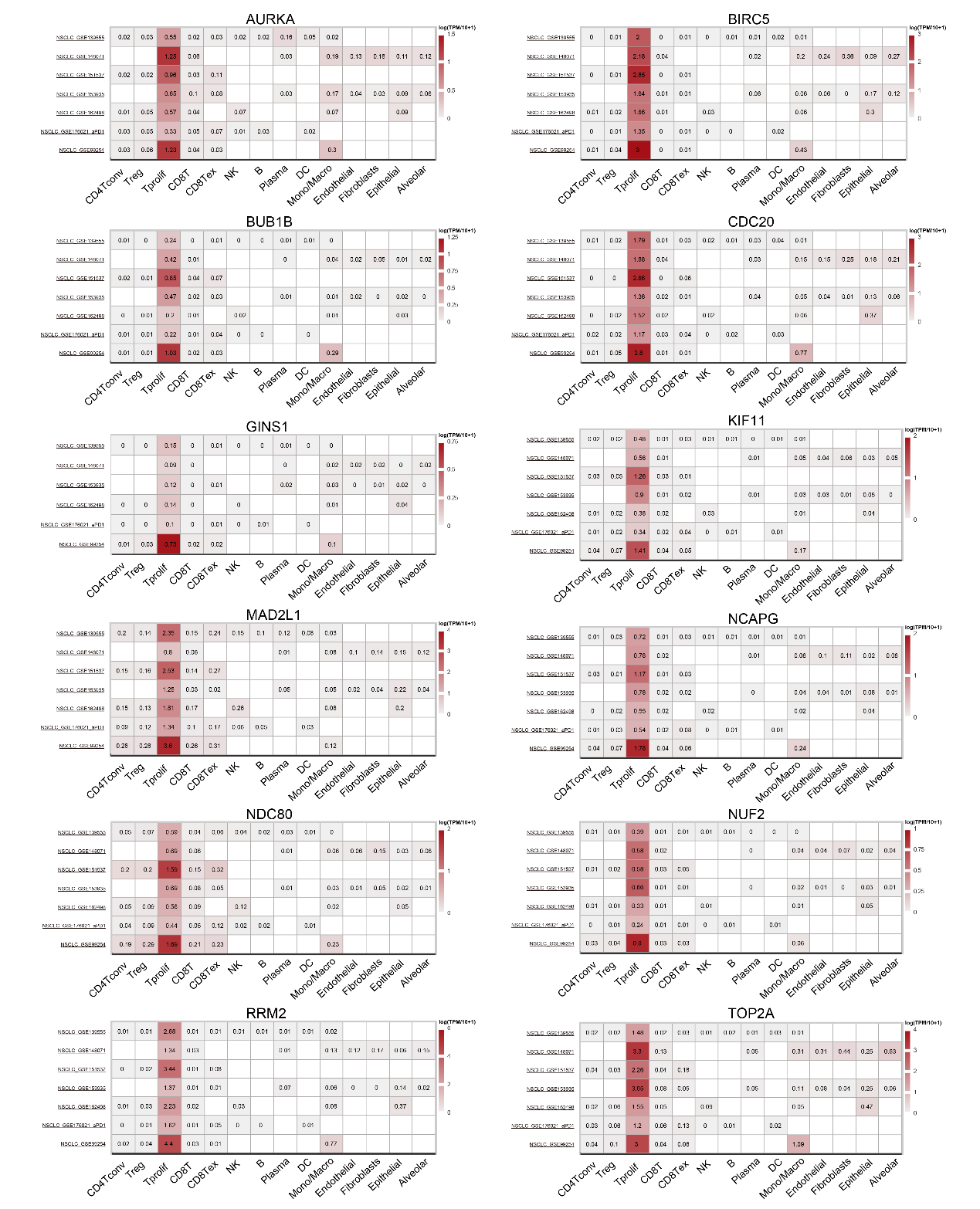


**Figure S19.** Single-cell expression levels of the CDSI core genes in different datasets.


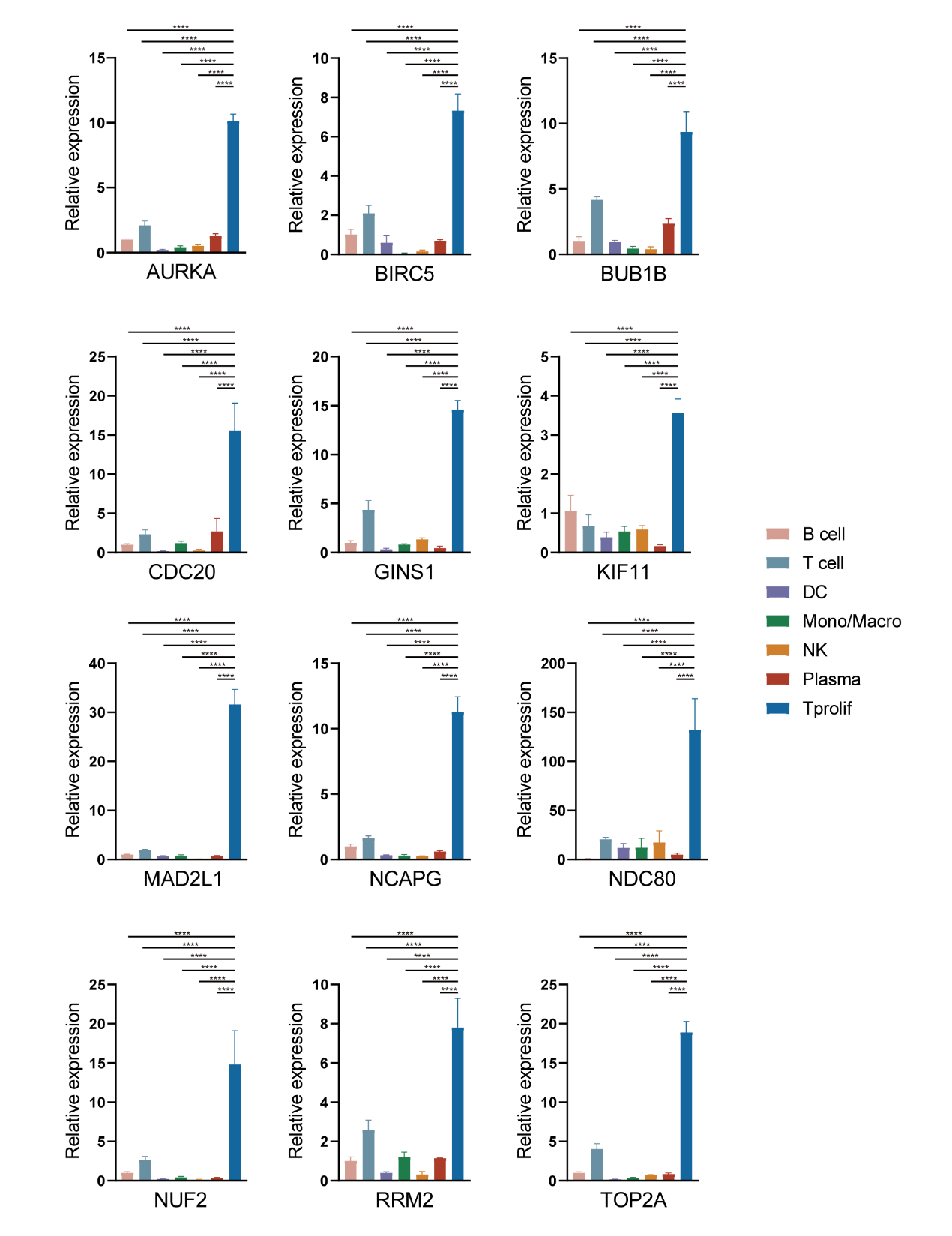


**Figure S20.** The expression levels of the CDSI core genes in different immune cells. The immune cells from tumor tissue were separated using flow cytometry. Relative expression levels of the CDSI core genes were measure by qRT-PCR. The p value was calculated using ordinary one-way ANOVA. ****p ≤ 0.0001.


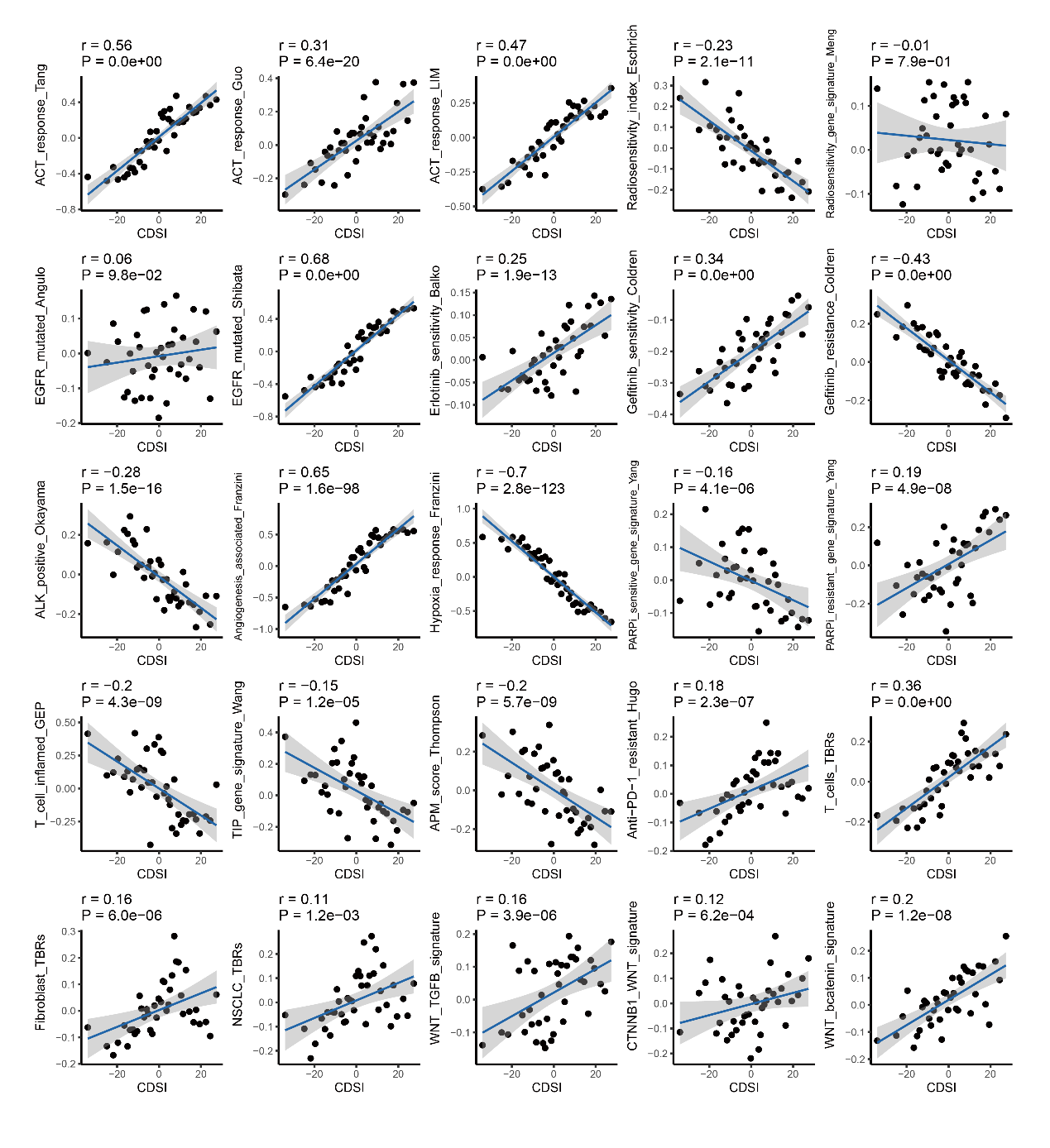


**Figure S21.** Association between CDSI and the therapeutic response signatures. Correlation between CDSI and the enrichment scores of therapeutic signatures calculated using GSVA. The p value was calculated using the Spearman correlation analysis.


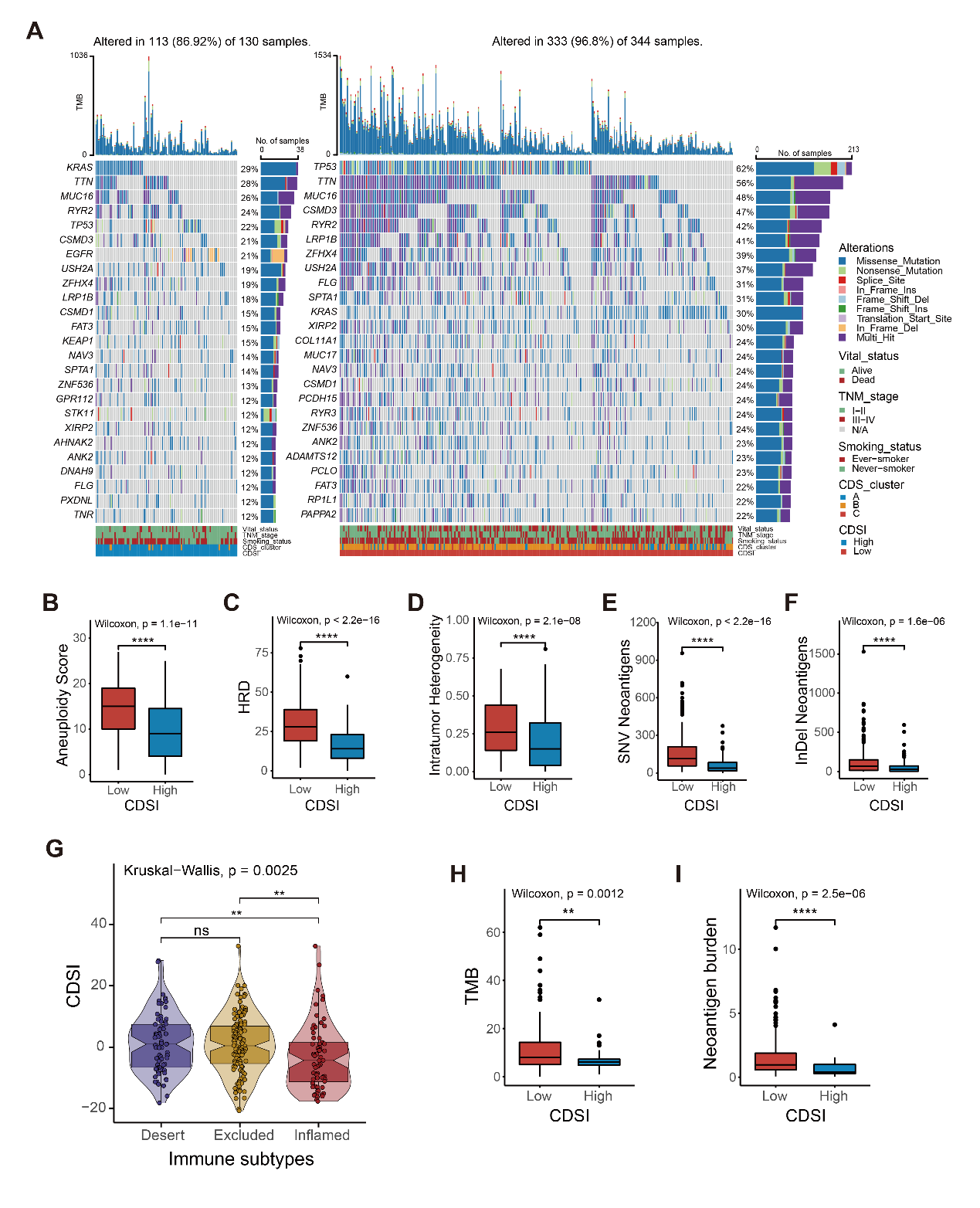


**Figure S22.** Association between CDSI and tumor somatic mutation. **A** The waterfall plot of tumor somatic mutation in the CDSI-high patients on the left (red) and CDSI-low patients on the right (blue). Individual patients are represented in each column. The upper barplot shows tumor mutation burden (TMB), the number on the right indicates the mutation frequency in each gene. The right barplot shows the proportion of each variant type. **B**–**F** Differences in (B–D) immunotherapy-related somatic variants, (E, F) neoantigen burdens between the CDSI-high and -low groups. **G** Difference in CDSI among immune phenotypes, including the inflamed, excluded, and desert immune type in the IMvigor210 cohort. **H, I** Differences in (**H**) tumor mutation burden (TMB) and (**I**) neoantigen burden between CDSI-high and -low groups in the IMvigor210 cohort. In **B–I**, the upper and lower ends of the boxes represent the interquartile range of values, the lines in the boxes represent median values, and black dots show outliers. P values were calculated using the Kruskal-Wallis test or Wilcoxon rank-sum test. Ns, not significant; **p ≤ 0.01; ****p ≤ 0.0001.
